# Proteome-Wide and Protein-Specific Multi-Epitope Vaccine Constructs Against the Rift Valley Fever Virus Outbreak Using Integrated Omics Approaches

**DOI:** 10.3389/fmicb.2022.921683

**Published:** 2022-05-31

**Authors:** Aqel Albutti

**Affiliations:** Department of Medical Biotechnology, College of Applied Medical Sciences, Qassim University, Buraydah, Saudi Arabia

**Keywords:** RVFV, proteins, proteome, MEVC, docking, immune factors

## Abstract

Rift Valley fever (RVF) is a viral disease caused by a member of the Bunyavirales family causing severe infections in humans. The RVF virus is an enveloped, negative-sense, single-stranded RNA virus that can infect both animals and humans. The symptoms associated with these infections span from minor (fever and headaches) to severe (meningoencephalitis and hemorrhagic fever syndrome) symptoms. Despite the outbreaks of the RVF virus being reported in different parts of the world, no effective therapy is available. Herein, the development of an efficient vaccine is critical for the control of infections associated with the RVF virus. Moreover, computational vaccine approaches are helpful in the design of specific, safe, and stable peptide-based designs when compared to the conventional methods of vaccine development. In this study, the whole proteome of the virus, comprising four proteins (NP, L, GP, and NSP), was screened to find putative vaccine epitope sequences (T cell, B cell, and HTL) specific for each protein. These shortlisted epitopes were then combined with flexible linkers to design protein-specific and proteome-wide immunogenic multi-epitope-based vaccine constructs. The results revealed that these multi-epitope vaccine constructs (MEVCs) are strongly antigenic and non-allergenic in nature. The efficacy of these constructs was further validated by docking with immune receptors, which revealed strong binding interactions with human TLR8. Using the MD simulation approach, the binding stability and residual flexibility of the best vaccine construct (proteome-wide) were confirmed, which revealed stable dynamic and favorable features. Furthermore, *in-silico* cloning and immune simulation analysis confirmed the expression and production of immune factors, that is, IgM, IgG, and IL-6, against the proposed vaccine designs. Additionally, 3D models of all the MEVC constructs have been developed and evaluated for potential immunization against the RVF virus. Finally, the proteome-wide vaccine candidate (MEVC-PW-RVFV) with the highest immune reinforcement potential provides new insights into the development of future vaccines against the emerging RVF virus.

## Introduction

Rift Valley fever (RVF) is a viral disease that can cause moderate to severe infections in livestock and humans. The Rift Valley fever virus (RVFV) belongs to the Bunyavirales family which mostly includes enveloped and negative-sense single-stranded RNA viruses. The main transmission routes of the RVF virus include coming in contact with the blood of infected animals and breathing in areas where the infected animals are butchered. However, it may also be transmitted by consuming raw milk of a sick animal or infected mosquito bites (Smithburn et al., 1949). This mainly includes spread by two mosquito species: Culex tritaeniorhynchus and Aedes vexans (Turell et al., 1996; Fontenille et al., 1998; Eifan et al., 2021). The viral infection can cause mild to severe symptoms in humans. The main symptoms include fever, headache, muscle pain, loss of vision, bleeding, and liver-related issues, which appear at different time intervals (Ikegami and Makino, 2011). Alarmingly, the condition may worsen to cause meningoencephalitis, hemorrhagic fever syndrome, or harm to the eyes in a rare number of cases (2%). People usually recover in 2–7 days following the onset of symptoms, and the disease may be fatal in about 1% of the cases (Gerdes, 2004).

The single-stranded RNA mainly constitutes the 11.5-kilobase tripartite genome of the RVF virus. This viral genome consists of two negative-sense (L and M segments) and one ambisense (S segment) RNAs. The L segment mainly encodes for L protein (viral polymerase), whereas the S segment encodes for the non-structural NS and nucleocapsid protein. On the other hand, the glycoprotein is encoded by the M segment. While numerous components of the viral RNA are involved in the pathogenesis, only the non-structural protein (coded by the S segment) has been demonstrated to show significant effects on the host. The inhibitory function of non-structural proteins in the host is mediated by a variety of factors, which includes the competitive inhibition of the synthesis of transcription factors (TFs) (Boshra et al., 2011). It mainly blocks the assembling of the transcription factor complex and leads to a suppressed antiviral response in the host (Boshra et al., 2011; Ikegami and Makino, 2011). These NS proteins also cause alterations in the activity of the double-stranded RNA-dependent protein kinase R which is essential to mediate the host's cellular antiviral reactions (Boshra et al., 2011).

The outbreaks of RVF, mostly common in African and Arab countries, are mostly associated with periods of excessive rainfall, which can be attributed to the boosted population of mosquitoes in the area. Previously, the disease was initially documented among animals in Kenya's Rift Valley, and the earliest reported outbreak occurred in 1931 causing fever in a large number of humans. Later, during the period 1974–1976, outbreaks of the RVF virus occurred in South Africa, where the first human death was reported (Van Velden et al., 1977; McMillen and Hartman, 2018). This was followed by an estimated 200,000 infections in Egypt causing 594 deaths (Arzt et al., 2010). Since then, outbreaks have occurred in Saudi Arabia and Yemen (2000), Sudan (2007), East Africa (2006–2007), South Africa (2010), (Nanyingi et al., 2015) Uganda (2016), Kenya (2018), and Mayotte (2018–2019) (Kenawy et al., 2018). Previously, numerous vaccination strategies have been deployed to prevent and cure a range of microbial infections while generating adaptive immune responses by delivering antigenic components to the immune system (Cunha-Neto et al., 2017). Although classical vaccine designs provide long-lasting protection, quick and large-scale production is not possible (Plotkin, 2009; Aslam et al., 2021). Herein, the advancement in using computational tools may reduce the cost and time required for developing peptide-based therapeutics significantly (Ul Qamar et al., 2021a,b).

Furthermore, designing vaccines based on computational techniques has proven its effectiveness, specificity, safety, and stability when compared to the conventional approaches to vaccine development (Ali et al., 2019; Khan et al., 2019a,b, 2021a,b; Naz et al., 2021). Previously, immuno-informatic strategies have been widely adopted for designing vaccines against several pathogens, including human immunodeficiency virus 1, ebola virus, herpes simplex virus 1 and 2, human norovirus, SARS-CoV, *Staphylococcus aureus, Shigella* spp., and *Candida auris* (Nosrati et al., 2019; Hasan et al., 2020; Ismail et al., 2021; Khan et al., 2022a,b). Previously, several strategies have been adopted to develop vaccines against the RVF virus, which include DNA vaccines (Lagerqvist et al., 2009), subunit vaccines (De Boer et al., 2010), virus replicon particle vaccines (Kortekaas et al., 2011), virus-like particles (VLPs) (Mandell et al., 2010), modified live vaccines (Habjan et al., 2008), virus vectored vaccines (Warimwe et al., 2016), inactivated vaccines (Pittman et al., 1999), and live attenuated vaccines (Morrill et al., 2013). However, still there is no available licensed vaccine against the RVF virus for human use. The current fundamental prediction measures and therapeutic modalities adopted against the RVF virus demand further development of effective vaccines.

In this scientific study, the proteome-wide epitopes were shortlisted against the different target proteins of the RVF virus. First, extensive analysis of T-cell, HTL, and B-cell epitopes was performed to predict the antigenic epitopes with potential utility as candidate sequences to design peptide-based vaccines against the RVF virus. This was followed by antigenicity, immunogenicity, and allergenicity profiling to shortlist potential antigenic peptides to be utilized in the design of protein-specific and proteome-wide vaccine designs against RVF virus. This research could pave the way for the development of a dynamic and efficient multi-epitope-based vaccine. The RVF whole proteome-derived highly antigenic putative epitope sequences combined with flexible linkers have been modeled and validated for the design of immunogenic peptide-based vaccines. Moreover, the *in-silico* clones and immunizing efficacy of each vaccine design have also been verified. Additionally, molecular docking and simulation analysis for the vaccine structure and human toll-like receptor 8 (TLR8) have also been performed. The current findings will aid in the development of potential peptide-based vaccine candidates against the RVF virus. However, further experimental processing is required to ensure the immune reinforcement potential of the proposed vaccine candidates.

## Methodology

**Figure 1:**
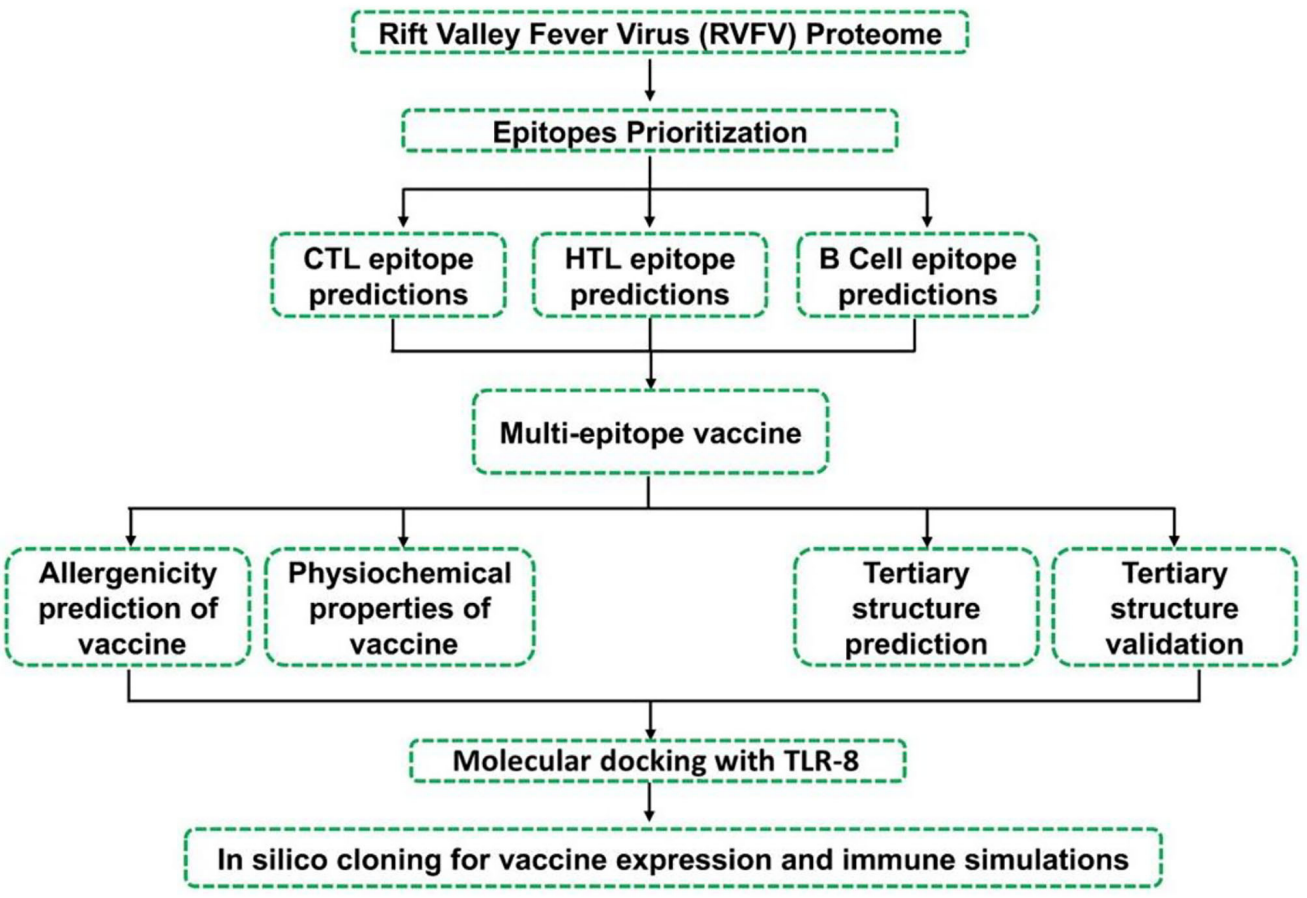
Showing adopted workflow including several steps used in the design of MEVC constructs against RVFV.

The methodological workflow of this study is presented in Figure 1.

### Epitope Prioritization and Immunogenic Potential Evaluation

The whole proteome and individual protein sequences were retrieved from the UniprotKB database (https://www.uniprot.org/) (Consortium, 2019). This was followed by putative epitope prediction for each target protein of the RVFV. First, T-cell epitopes were predicted for the individual proteins (NP, L, GP, and NSP) using the NetCTL1.2 server (http://www.cbs.dtu.dk/services/NetCTL/) at 0.75 thresholds (Larsen et al., 2007). These predictions were based on peptide binding to MHC-I, proteasomal C-terminal cleavage score, and transport efficiency of transporter associated with antigen processing (TAP). An artificial neural network was used to calculate peptide binding to MHC-I and proteasomal C-terminal cleavage scores, whereas the TAP score was calculated by using the weight matrix (Larsen et al., 2007; Khan et al., 2019a). Similarly, the prediction of HTL epitopes was performed on the online server Immune Epitope Database (IEDB) (http://www.iedb.org/) (Vita et al., 2019), considering a reference set of seven human HLAs by default. HTL epitopes were predicted against a reference set of HLAs under default parameters for maximal population coverage and more accurate/reliable epitopes for vaccine designing. HLA alleles are highly polymorphic throughout populations, and no comprehensive screening system exists to determine whether there is a link between the incidence of RVFV and the susceptibility/resistance of different HLA alleles. As such, it is logical to employ reference sets of HLA alleles with the greatest population coverage in such disorders (Ahmad et al., 2020). The server assigns IC50 values calculated for the HTL epitopes that are inversely proportional to their binding affinity for MHC-II molecules. IC50 scores of <50 nM represent a high binding affinity. The IC50 value <500 nM corresponds to intermediate binding affinity, while <5,000 nM corresponds to the low binding affinity of epitopes toward MHC-II. The binding affinity of predicted epitopes toward the MHC-II is inversely related to the percentile rank. IFN-epitope server (http://crdd.osdd.net/raghava/ifnepitope/) was employed to predict the interferon-gamma inducing potential of HTL epitopes that have passed the previous parameters (Dhanda et al., 2013). It is based on motif and SVM hybrid algorithm approaches to predict the interferon-producing capability of an epitope. The server assigned the SVM (support vector machine) score for each input epitope. HTL epitopes that can induce IFN-gamma response and fulfill the set parameters were selected for multi-epitope vaccine construction. B-cell epitopes are recognized and bound by receptors found on the surface of B cells. In the antibody production pathway of the host, B-cell epitopes play a significant role. An online web tool ABCPreds (http://crdd.osdd.net/raghava/abcpred/) (EL-Manzalawy et al., 2008) is a new method for predicting linear B-cell epitopes and uses the kernel method. SVM is a well-known component of kernel techniques, which consists of many algorithms used for pattern analysis. The output performance of the ABCPreds server (AUC = 0.75) is based on the support vector machine in combination with amino acid pair antigenicity (AUC = 0.7). Finally, all the selected epitopes (T cell, B cell, and HTL) were further analyzed for antigenic potential using the Vaxijen server (Doytchinova and Flower, 2007). This server predicts the antigenic score wholly based on the query amino acid sequence physicochemical properties as a substitute for using sequence alignment algorithms. Vaxijen precision for antigenicity is quite reliable (70–89%). Only the antigenic epitopes that scored higher than 0.4 were selected for further evaluation. The epitopes fulfilling the above-mentioned parameters were subjected to allergenic potential analysis using the Algpred2.0 server (https://webs.iiitd.edu.in/raghava/algpred2/) (Sharma et al., 2021). Algpred2.0 is an improved version of Algpred sever developed in 2006; numerous features have been incorporated to improve the performance of the method. The server calculates the allergenicity potential of a given amino acid sequence with a specificity and sensitivity of 95 and 93%, respectively, at a default cut-off score of 0.3.

### Epitope Conservancy and Population Coverage Analysis

The epitopes included in the final vaccine designs were also subjected to conservancy analysis. All the three classes of epitopes (T cell, HTL, and B cell) were screened by using sequences of each target protein using BLASTP (https://blast.ncbi.nlm.nih.gov/Blast.cgi) (Mahram and Herbordt, 2010) tool against a wide range of species. The aligned sequences were also analyzed through the Clustal Omega (https://www.ebi.ac.uk/Tools/msa/clustalo/) (Sievers and Higgins, 2014) package to check the conservancy of the shortlisted epitopes. Moreover, population coverage analysis was also performed using the IEDB population coverage analysis tool (http://tools.iedb.org/population/) (Bui et al., 2006) by choosing the Class II separate option to decipher the worldwide distribution of each HTL epitope.

### Molecular Docking of HTL Epitopes With Corresponding HLAs

The inclusion of the putative HTL epitope vaccine was preceded by molecular docking analysis through Hawk-dock server with respective human leukocyte antigens. There were no available structures for the three HLAs (HLA-DRB4^*^01:01, HLA-DRB3^*^01:01, and HLA-DRB1^*^07:01), which showed higher binding affinity toward the shortlisted HTL epitopes. These structures were modeled by using the Robetta server, and the obtained 3D structures of these human leukocyte antigens corresponding to the selected HTL epitopes were used in the docking analysis. Moreover, all the HTL epitopes were modeled using the PEPstrMOD server (Singh et al., 2015). This was followed by receptor (HLAs) and the ligand (HTL epitopes) docking by utilizing the HawkDock server (http://cadd.zju.edu.cn/hawkdock/) (Weng et al., 2019) to calculate binding scores for each docking complex.

### Vaccine Construction, Structural Modeling, and Validation

All the selected epitopes after immunogenic potential evaluation were subjected to the process of vaccine construction. Different multi-epitope-based vaccine constructs (protein-specific and proteome-wide) were designed against RVFV. This was achieved through the use of different linkers (AAY, GPGPG, and KK) that were used to join different epitope sequences in the final constructs. Additionally, an adjuvant human beta-defensin 2 (HbD-2) was linked at the N-terminal end to improve the stability, folding, and immune response. Furthermore, the 3D structures for each designed MEVC were also modeled using the Robetta server. The physicochemical properties were also evaluated for each of the designed vaccines by using Expasy Tool. Additionally, all the finalized MEVCs were also evaluated for antigenicity and allergenicity status.

### *In-silico* Cloning and Codon Optimization

All the vaccine construct sequences were further reverse-transcribed through the use of the jCat server to acquire the optimized DNA sequences (Grote et al., 2005). The Codon Adaptation Index (CAI) values and GC content for each of the vaccine construct sequences were determined (**Table 6**). Each obtained DNA sequence was then inserted into a protein expression plasmid vector (PET 28 a +). For this purpose, we selected two restriction enzyme (*Xho1* and *EcoR1*) sites and obtained the cloning designs for each of the designed MEVCs. The software package SnapGene was utilized to perform the *in-silico* cloning and acquire the final plasmid maps.

### Molecular Docking and Simulation of MEVCs With Human TLR8 and HLAs With HTL Epitopes

All the designed MEVCs were subjected to molecular docking with human TLR8 and HLAs with HTL epitopes by using the HDOCK server (Yan et al., 2017). The server utilizes a hybrid algorithm for template-based or template-free docking analysis depending on the input to predict interactions between ligand and receptor. The server possesses a significant ability to process the PDB structures of the interacting molecules. This includes the utility of hybrid strategies like protein–protein binding sites and small-angle X-ray scattering information during the process of docking. A blind docking approach (Muneer et al., 2021) was employed where the binding site residues were not defined to test the accuracy of the server. No restraint was set, and each molecule was allowed for conformational optimization. Clusters were generated, and the best representative clusters were grouped and reported as those with the best docking orientation. The conformation with the lowest energy was selected for further analysis. The best vaccine candidate, based on proteome-wide analysis, was subjected to MD simulation using FF19SB of AMBER20 (Pearlman et al., 1995; Case et al., 2005). Two steps minimization followed by heating and equilibration was performed. Each step of minimization included 6,000 runs and heating at 300 K and 1 atm pressure. Equilibration was executed for 10 ns, and with a time step of 0.02 ps, a 50 ns production run was performed for the TLR8–vaccine complex. Steepest descent and conjugate gradient algorithm were used. For the processing of post-simulation trajectories, CPPTRAJ and PTRAJ were used (Roe and Cheatham, 2013).

### Immune Simulation

*In silico* immune simulation was carried out to predict the real-life immune system response to the multi-epitope vaccine constructs using the C-ImmSim server (Rapin et al., 2010). This simulant employs machine learning (ML) and position-specific scoring matrix (PSSM) for the prediction of the immune system and epitope interactions, respectively. The least suggested gap between the first and second doses of most of the vaccines currently being used is 4 weeks (Castiglione et al., 2012). Three injections, each containing 1,000 units of the vaccine, were given 4 weeks apart for our immune simulation. For calculating simulation durations, the C-ImmSim server employs a time-step scale. Each time step on this scale corresponds to 8 h in real life. The total number of time steps for simulation was customized to 1,050, with the injection points set at time steps 1, 84, and 168, respectively. The remaining parameters were left at their default values.

## Results and Discussion

### Sequences Retrieval

The whole proteome sequence of Rift Valley fever virus (RVFV) having proteome ID “**UP000135029”** and individual amino acid sequences of four proteins, namely, nucleoprotein, replicase, non-structural protein, and glycoprotein, were downloaded from UniProt. The accession details of each protein of the Rift Valley fever virus evaluated for further screening of putative T-cell, B-cell, HTL, and IFN epitopes are given in Table 1.

**Table 1:** Accession information and other details of assessed proteins.

**Accession ID**	**Entry name**	**Protein name**	**Organism**	**Amino acids length**
A2T003	A2T003_RVFV	Nucleoprotein	RVFV	245
B6S1Y8	B6S1Y8_RVFV	Replicase	RVFV	2,092
F5BUA8	F5BUA8_RVFV	Non-structural protein NS-S	RVFV	265
B1NHK9	B1NHK9_RVFV	Glycoprotein	RVFV	1,197

### Prioritization of Putative Vaccine Epitopes

All the protein sequences of the RVFV proteome were first screened for putative vaccine epitopes. The selected final vaccine epitopes after immunogenic potential evaluation were included in the final vaccine design. Moreover, the characterization of peptides recognized by the immune system is a pre-requisite for peptide-based vaccine designs.

#### T-cell Epitopes

The recognition of antigenic epitopes by T cells is vital for the induction of immune response. This prediction of epitopes recognized by T cells helps to combat the evading pathogen and stop them from developing infections. Moreover, the prediction of potential T-cell epitopes based on binding specificity through *in-silico* approaches offers wide utilities. First, the total number of T-cell epitopes was predicted for each protein of RVFV. The number of identified T-cell epitopes for nucleoprotein was 6. Similarly, a total of 70, 1, and 25 T-cell epitopes were predicted for replicase, NS-S, and GP, respectively. All the identified T-cell epitopes were further screened to select the putative vaccine epitopes among all. After mapping, selected T-cell epitopes with high immunogenic potential (Table 2) were included in further vaccine design.

**Table 2:** Details of total identified T-cell epitopes and shortlisted ones for the vaccine design against each target protein of RVFV.

**Target protein**	**Predicted T cell epitopes**	**T-cell epitope**
Nucleoprotein	6	TMDGLSPAY AVMAAAQAY
Replicase	70	DSAAMDASY RSSPPDSEY
Non-structural protein NS-S	1	LLPGFDLMY
Glycoprotein	25	LSCREGQSY KTILLICLY

#### B-cell Epitopes

The mapping of B-cell epitopes also plays a key role in the induction of robust immune responses. This is due to the important role of B cells in regulating immune response through the production of antibodies against the invading pathogen. This B-cell response is triggered upon the identification of epitope sequences presented by the antigen. Moreover, the identification of highly immunogenic B-cell epitopes is a pre-requisite in peptide-based vaccine designs. Second, the total number of B-cell epitopes was predicted for each protein of RVFV. The number of identified B-cell epitopes for nucleoprotein was 29. Similarly, a total of 223, 27, and 118 B-cell epitopes were predicted for replicase, NS-S, and GP, respectively. All the identified B-cell epitopes were further screened to select putative vaccine epitopes among all. After mapping, selected B-cell epitopes with high immunogenic potential (Table 3) were included in further vaccine design.

**Table 3:** Details of total identified B-cell epitopes and shortlisted ones for the vaccine design against each target protein of RVFV.

**Target protein**	**Predicted b-cell Epitopes**	**B-cell epitope**
Nucleoprotein	29	EWLPVTGTTMDGLSPA FGLVDSNGKPSAAVMA
Replicase	223	DLHILSYTASDNDLSP DGSISWGGMFNPFSGR
Non-structural protein NS-S	27	LPSMMGRNNWIPVVPP SLMLRSSLPSMMGRNN
Glycoprotein	118	EHKGQYKGTMDSGQTK RVLKCLKIAPRKVLNP

#### HTL Epitopes

Similarly, the identification of HTL epitopes also has a crucial role in the development of a protective immune response against invading pathogens. They play a key role in B-cell activation and maturation. This inclusion of HTL epitopes in the peptide-based vaccine constructs helps in the development of safe and effective therapies against human pathogens. Finally, the total number of HTL epitopes was predicted for each protein of RVFV. The number of identified B-cell epitopes for nucleoprotein was 1,617. Similarly, a total of 14,546, 1,757, and 8,281 B-cell epitopes were predicted for replicase, NS-S, and GP, respectively. All the identified HTL epitopes were further screened to select putative vaccine epitopes among all. After mapping, HTL epitopes based on IFN response induction potential and population coverage analysis, following selected epitopes (Table 4), were included in further vaccine design.

**Table 4:** Details of total identified HTL epitopes and shortlisted ones for the vaccine design against each target protein of RVFV.

**Target protein**	**Total number of predicted HTL epitopes**	**Selected IFN- positive HTL epitopes**	**Organism**	**Amino acids length**	**Population coverage (%) worldwide %**
Nucleoprotein	1,617	AFGLVDSNGKPSAAV VINPNLRGRTKEEVA	RVFV	245	81.38
Replicase	14,546	EAEVPWAFKGKTYLE SSTDEELGKTERELL	RVFV	2,092	81.38
Non-structural protein NS-S	1,757	EIAHVQCVRLLQAAR MYEIAHVQCVRLLQA	RVFV	265	81.38
Glycoprotein	8,281	ALGNPAPIPRHAPIP ACASGVCVTGSQSPS	RVFV	1,197	81.38

### Epitope Conservancy Analysis

All the putative vaccine epitopes were also analyzed for conservancy analysis. This included the screening of epitope (T cell, HTL, and B cell) sequences shortlisted against each target protein during alignment with sequences from other species. The results generated by the Clustal Omega (https://www.ebi.ac.uk/Tools/msa/clustalo/) were then collected to predict the conservancy of each epitope. This analysis revealed the presence of most of the epitopes included for vaccine construction, while following higher sequence conservancy across species. However, few epitopes were also found to be less conserved. All the data collected for each epitope has been included in a separate Supplementary File for each epitope (B cell, T cell, and HTL), as shown in Supplementary Figures 1A–W.

### HTL Epitope-HLA Molecular Docking

Moreover, before the inclusion of shortlisted HTL epitopes in the final vaccine designs, HTL epitopes were also subjected to docking analysis with corresponding human alleles having strong binding affinities. These HTL epitopes were also assessed for putative interactions with the corresponding HLAs, and the binding scores for each epitope were also evaluated. The higher binding energies obtained after the docking analysis of studied HTL epitopes with their corresponding HLAs were indicative of their potential ability to be utilized in vaccine designs. The details including peptide sequences, corresponding HLAs, and binding energies for each of the docking complexes are given in Table 5.

**Table 5:** Corresponding HLA with higher binding affinity and docking scores obtained for each HTL epitope of the target RVFV proteins.

**Target protein**	**Selected IFN- positive HTL epitopes**	**Corresponding HLA**	**Binding energy scores**
Nucleoprotein	AFGLVDSNGKPSAAV VINPNLRGRTKEEVA	LA-DRB4*01:01 HLA-DRB4*01:01	−56.23 −61.42
Replicase	EAEVPWAFKGKTYLE SSTDEELGKTERELL	HLA-DRB4*01:01 HLA-DRB3*01:01	−49.88 −56.75
Non-structural protein NS-S	EIAHVQCVRLLQAAR MYEIAHVQCVRLLQA	HLA-DRB3*01:01 HLA-DRB3*01:01	−39.58 −71.22
Glycoprotein	ALGNPAPIPRHAPIP ACASGVCVTGSQSPS	HLA-DRB1*07:01 HLA-DRB3*01:01	−54.35 −61.33

**Figure 2:**
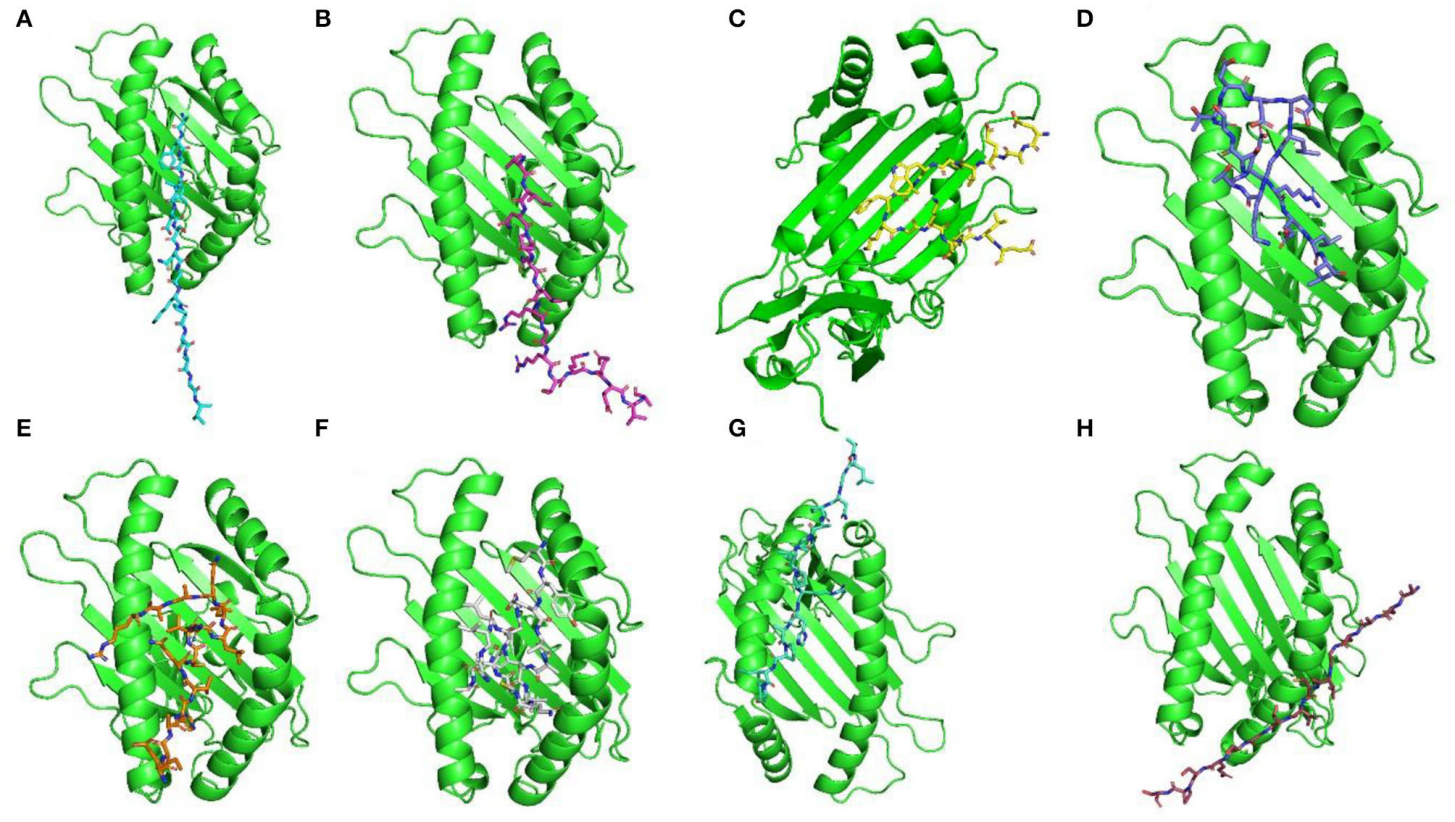
Docking complexes of selected HTL epitopes with corresponding HLAs. Here **(A)** shows AFGLVDSNGKPSAAV + HLA-DRB4*01:01, **(B)** shows VINPNLRGRTKEEVA + HLA-DRB4*01:01, **(C)** shows EAEVPWAFKGKTYLE + HLA-DRB4*01:01, **(D)** shows SSTDEELGKTERELL + HLA-DRB3*01:01, **(E)** shows EIAHVQCVRLLQAAR + HLA-DRB3*01:01, **(F)** shows MYEIAHVQCVRLLQA + HLA-DRB3*01:01, **(G)** shows ALGNPAPIPRHAPIP + HLA-DRB1*07:01, and **(H)** shows ACASGVCVTGSQSPS + HLA-DRB3*01:01.

For docking complexes of HTL epitopes with the corresponding HLAs, the total binding free energy was −56.23 kcal/mol for HLA-DRB4^*^01:01-AFGLVDSNGKPSAAV, −61.42 kcal/mol for HLA-DRB4^*^01:01-VINPNLRGRTKEEVA, −49.88 kcal/mol for HLA-DRB4^*^01:01-EAEVPWAFKGKTYLE, −56.75 kcal/mol for HLA-DRB3^*^01:01- SSTDEELGKTERELL, −39.58 kcal/mol for HLA-DRB3^*^01:01-EIAHVQCVRLLQAAR, −71.22 kcal/mol for HLA-DRB3^*^01:01-MYEIAHVQCVRLLQA, −54.35 kcal/mol for HLA-DRB1^*^07:01-ALGNPAPIPRHAPIP, and −61.33 kcal/mol for HLA-DRB3^*^01:01- ACASGVCVTGSQSPS. The HTL–HLA docking complexes are shown in Figure 2.

### Vaccine Constructs

**Figure 3:**
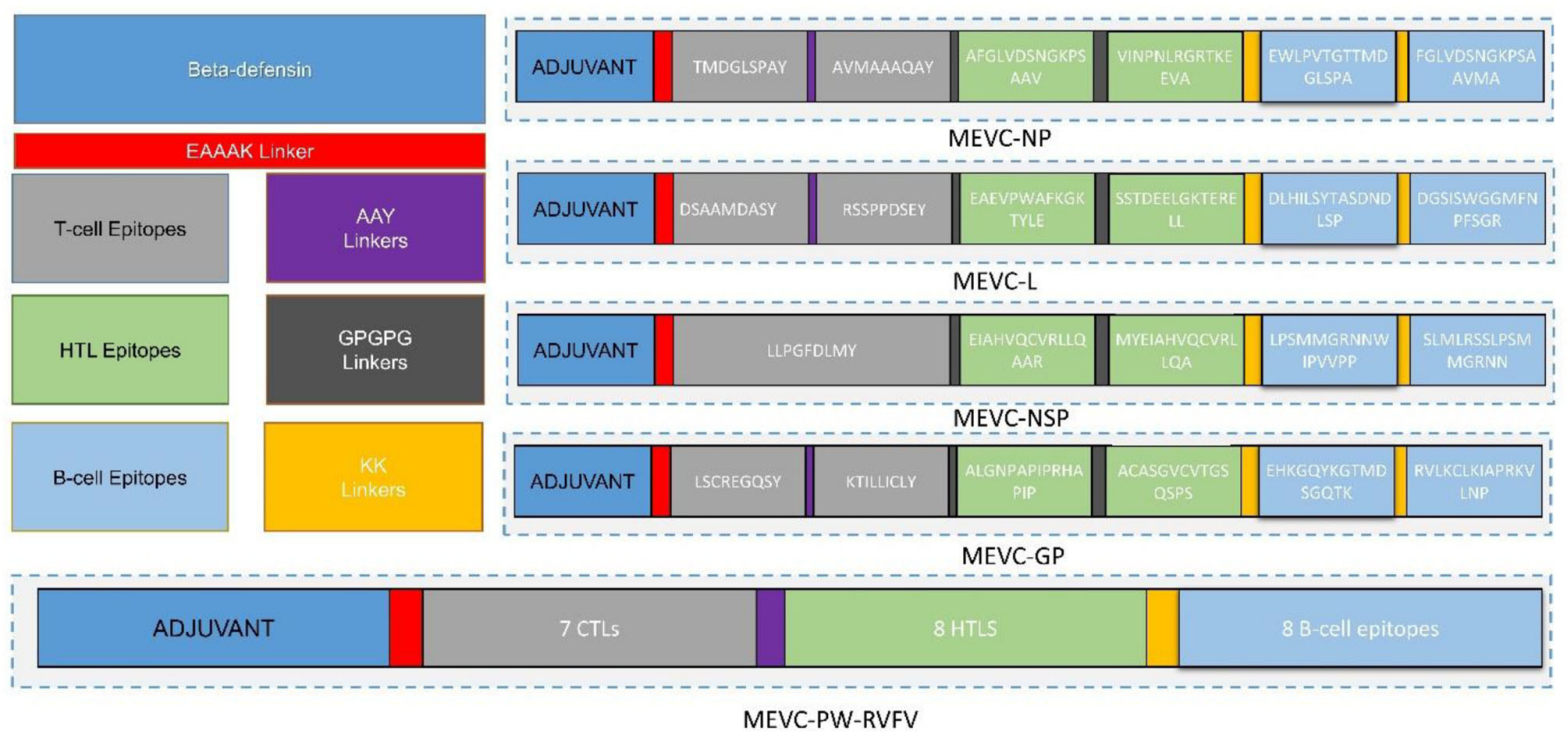
Topological organization of MEVCs designed against RVFV. This includes the protein-specific and proteome-wide epitopes joined with concerned linkers in the anti-RVFV vaccine designs.

The use of computational vaccinology approaches has accelerated the design of protective vaccines with increased safety and efficacy. These highly specific vaccine constructs based on the inclusion of immunogenic epitopes are vital for the induction of robust immune response. These vaccine constructs are highly immunogenic and non-allergenic. Moreover, the use of proper adjuvant and linkers are also required for the proper designing of the final vaccine constructs (Ahmad et al., 2021; Rehman et al., 2021; Ul Qamar et al., 2021c). Herein, all the putative epitopes after screening were subjected to the construction of multi-epitope-based vaccine constructs. A total number of four vaccine constructs were designed against each target protein of RVFV. This was achieved through joining the different putative epitopes (Tables 2–4) with the help of different linkers, that is, EAAK, AAY, GPGPG, and KK (Figure 3). Additionally, a multi-epitope-based vaccine was also designed by targeting the whole proteome of RVFV. This included epitopes from all the four proteins of RVFV joined together in the final vaccine construct named PW-RVFV. All the constructed vaccine sequences were further evaluated for immunogenicity potential. This was performed through analysis of each vaccine sequence on the basis of antigenicity and allergenicity status. Additionally, a negative control sequence constructed based on the whole proteome was also evaluated in the study. The details of all the designed multi-epitope-based vaccine constructs along with their immunogenicity status are given in Table 6.

**Table 6:** Details of the final vaccine constructs and their immunogenic potential designed against each target protein of RVFV.

**Vaccine name**	**Target protein**	**Protein specific/Proteome wide MEVC constructs**	**Number of amino acids**	**Antigenicity score**	**Antigenicity status**	**Allergenicity**
MEVC-NP	Nucleoprotein	MRVLYLLFSFLFIFLMPLPGVFGGIGDPVTCLKSGAICHPVFCPRRYKQIGTCGLPGTKCCKKPEAAKTMDGLSPAYAAYAVMAAAQAYGPGPGAFGLVDSNGKPSAAVGPGPGVINPNLRGRTKEEVAKKEWLPVTGTTMDGLSPAKKFGLDSNGKPSAAVMA	165	0.6459	ANTIGEN	NON-ALLERGEN
MEVC-L	Replicase	MRVLYLLFSFLFIFLMPLPGVFGGIGDPVTCLKSGAICHPVFCPRRYKQIGTCGLPGT KCCKKPEAAKDSAAMDASYAAYRSSPP DSEYGPGPGEAEVPWAFKGKTYLEGPGPGSSTDEELGKTERELLKKDLHILSYTASDNDLSPKKDGSISWGGMFNPFSGR	165	0.6182	ANTIGEN	NON-ALLERGEN
MEVC-NSP	Non-structural protein NS-S	MRVLYLLFSFLFIFLMPLPGVFGGIGDPVTCLKSGAICHPVFCPRRYKQIGTCGLPGT KCCKKPEAAKLLPGFDLMYGPGPGEIAHVQCVRL LQAARGPGPGMYEIAHVQCVRLLQAKKLPSMMGRNNWIPVVP PKKSLMLRSSLPSMMGRNN	153	0.6787	ANTIGEN	NON-ALLERGEN
MEVC-GP	Glycoprotein	MRVLYLLFSFLFIFLMPLPGVFGGIGDPVTCLKSGAICHPVFCPRRYKQIGTCGLPGTKCC KKPEAAKLSCREGQSYAAYKTILLICLYGPGPGALGNPAPIPRHAPIPGPGPGACASGVCVTGSQSPSKKEHKGQYKGTMDSGQTKKKRVLKCLKIAPRKVLNP	165	0.5142	ANTIGEN	NON-ALLERGEN
MEVC-PW-RVFV	Whole Proteome	MRVLYLLFSFLFIFLMPLPGVFGGIGDPVTCLKSGAICHPVFCPRRYKQIGTCGLPGTKCCKKPEAAKTMDGLSPAYAAYAVMAAAQAYAAYDSAAMDASYAAYRSSPPDSEYAAYLLPGFDLMYAAYLSCREGQSYAAYKTILLICLYGPGPGAFGLVDSNGKPSAAVGPGPGVINPNLRGRTKEEVAGPGPGEAEVPWAFKGKTYLEGPGPGSSTDEELGKTERELLGPGPGEIAHVQCVRLLQAARGPGPGMYEIAHVQCVRLLQAGPGPGALGNPAPIPRHAPIPGPGPGACASGVCVTGSQSPSKKEWLPVTGTTMDGLSPAKKFGLVDSNGKPSAAVMAKKDLHILSYTASDNDLSPKKDGSISWGGMFNPFSGRKKLPSMMGRNNWIPVVPPKKSLMLRSSLPSMMGRNNKKEHKGQYKGTMDSGQTKKKRVLKCLKIAPRKVLNP	453	0.6437	ANTIGEN	NON-ALLERGEN
MEVC-PW-NEG	Whole Proteome	FAAQAVDRNEIEQWVAAQAVDRNEIEQWVRSACSSDLWATDEDLYWTCATSDDARKWNQGLFCQSSEDDGSKLKTRNRPGKGHNYIDGMTANAYCSHANGSGIVQVAHCPPQDPCLVHGCVPEVEEEFMYSCDGDFCQSSEDDGSKLKTKDLFCQSSEDDGSKLKANAYCSHANGSGIVQVAHCPPQDPCLVHGCRPGKGHNYIDGMTQECQSSEDDGSKLKTKM	225	0.12	NON-ANTIGEN	NON-ALLERGEN

*For these predictions, VaxiJen and Algpred servers were used*.

The peptide-based vaccine constructs composed of different immune epitopes are targeted by antibodies in a conformation-dependent manner when subunit peptide vaccines are delivered to the human body (Black et al., 2010). This is accomplished by the interaction of epitopes carried on the antigenic residues whose side chains come in direct contact with the antibody-combining site. Antibody recognition is dependent on the globular fold, at least in the region of the epitope, because larger structural epitopes are often conformation-dependent and incorporate residues from many secondary structural components (Wang et al., 2010) While the stretch of amino acids does not need to be in a specific conformation to be identified by the antibody, they are normally encountered by local antibody binding. For T-cell responses as well, some regions of an antigen may result in a more efficient expansion of T cells than others. Issues of immunodominance are an important consideration for any vaccine design strategy, particularly for peptide vaccines that focus on only a single or a few critical epitopes (Malonis et al., 2019). Selection of highly antigenic epitopes is a pre-requisite that allows induction of strong immune responses through the administration of a peptide-based vaccine.

### Physicochemical Information

It is vital to evaluate the numerous physicochemical properties of the designed MEVCs to ensure the safety and efficacy of the vaccine designs. Furthermore, all the constructed vaccines were evaluated for the characterization of different physicochemical properties. This was performed to ensure the experimental feasibility of the constructed vaccines. The analysis was performed for each of the individual vaccine constructs designed for target proteins, that is, MEVC-NP, MEVC-L, MEVC-NSP, and MEVC-GP. Moreover, the same analysis was also performed for the whole proteome-based design MEVC-PW-RVFV. The results demonstrated different approximate ranges for each analyzed parameter, including GC content (53–55), CAI value (0.94–1), molecular weight (16–47 kd), theoretical pI (7.56–9.84), and others, for the designed MEVCs. On the other hand, all the parameters were also analyzed for the negative control (MEVC-PW-NEG), and the results were compared. A significant diversion in the multiple analyzed parameters was observed. The details of different physicochemical information extracted for each of the MEVC constructs are given in Table 7. The data indicated that the negative control does not possess favorable physicochemical properties when compared to the designed vaccine candidates. These results also reflected the hydrophilic and stable nature of all the designed MEVCs except the negative control.

**Table 7:** Details of the physicochemical information associated with each of the final vaccine constructs against RVFV.

**Vaccine name**	**GC-content**	**CAI-value**	**Molecular weight**	**Theoretical pI**	**Neg charged residues (Asp + Glu):**	**Positively charged residues (Arg + Lys)**	**Half-life**	**Total number of atoms**	**Aliphatic index**	**Hydropathicity (GRAVY)**
MEVC-NP	54.34	0.95	17094.14	9.41	9	18	Mammals >30 h Yeast >20 h *E. coli* >10 h	2,419	77.52	0.15
MEVC-L	53.13	1	17795.4	7.56	18	19	Mammals >30 h m Yeast >20 h *E. coli* >10 h	2,479	65.09	−0.281
MEVC-NSP	55.11	0.95	16742.29	9.86	5	19	Mammals >30 h Yeast >20 h *E. coli* >10 h	2,385	90.46	0.202
MEVC-GP	54.54	0.96	17516.98	9.77	5	24	Mammals >30 h Yeast >20 h *E. coli* >10 h	2,500	79.82	−0.021
MEVC-PW-RVFV	54.45	0.94	47753.47	9.43	31	53	Mammals >30 h Yeast >20 h *E. coli* >10 h	6,717	72.23	−0.203
MEVC-PW-NEG	48	0.58	24685.04	4.75	36	20	Mammals 1.1 h Yeast 3 min *E. coli* 2 min	3,311	51.20	−0.743

*ProtParam server was used for the prediction of physicochemical properties*.

### Structural Modeling and Validation of the MEVCs

**Figure 4:**
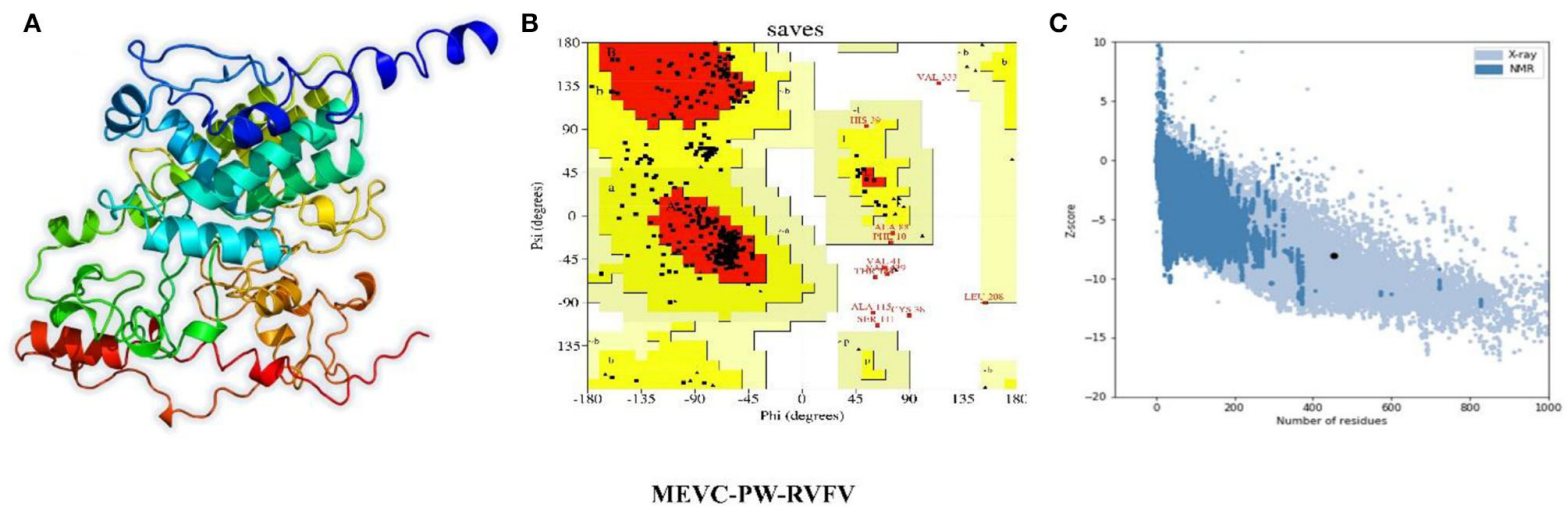
Modeled 3D structures of MEVC designed against the whole proteome of RVFV. Here, **(A)** represents the 3D modeled structures of MEVCs, **(B)** represents the Ramachandran plot analysis, and **(C)** represents the ProSA-Web analysis of the 3D modeled structure.

Next, the linear vaccine constructs were subjected to 3D modeling for the depiction of the MEVC structures. By utilizing the structural modeling package Robetta, all the MEVC structures were modeled based on the amino acid sequences. This was performed by submission of these sequences to the online server using the Robetta-Fold option, and the obtained structures were refined by using PyMOL. The different 3D models generated for the structural model of each MEVC are given in Figure 4. Moreover, all the modeled structures for each protein-specific MEVC (Supplementary Figure 2) were also validated through Ramachandran and ProSA-Web analysis (Supplementary Figures 3, 4). The 3D structural modeling and validations were also performed for the negative control, with more loops in the structure and deviated values (Supplementary Figure 5) reflecting the validity of the MEVCs designed in the study. The structure determined for the negative control reported more loops and unfolded topology of the protein, which consequently demonstrated the dysfunctionality of the negative control.

### *In-silico* Cloning of the MEVCs

**Figure 5:**
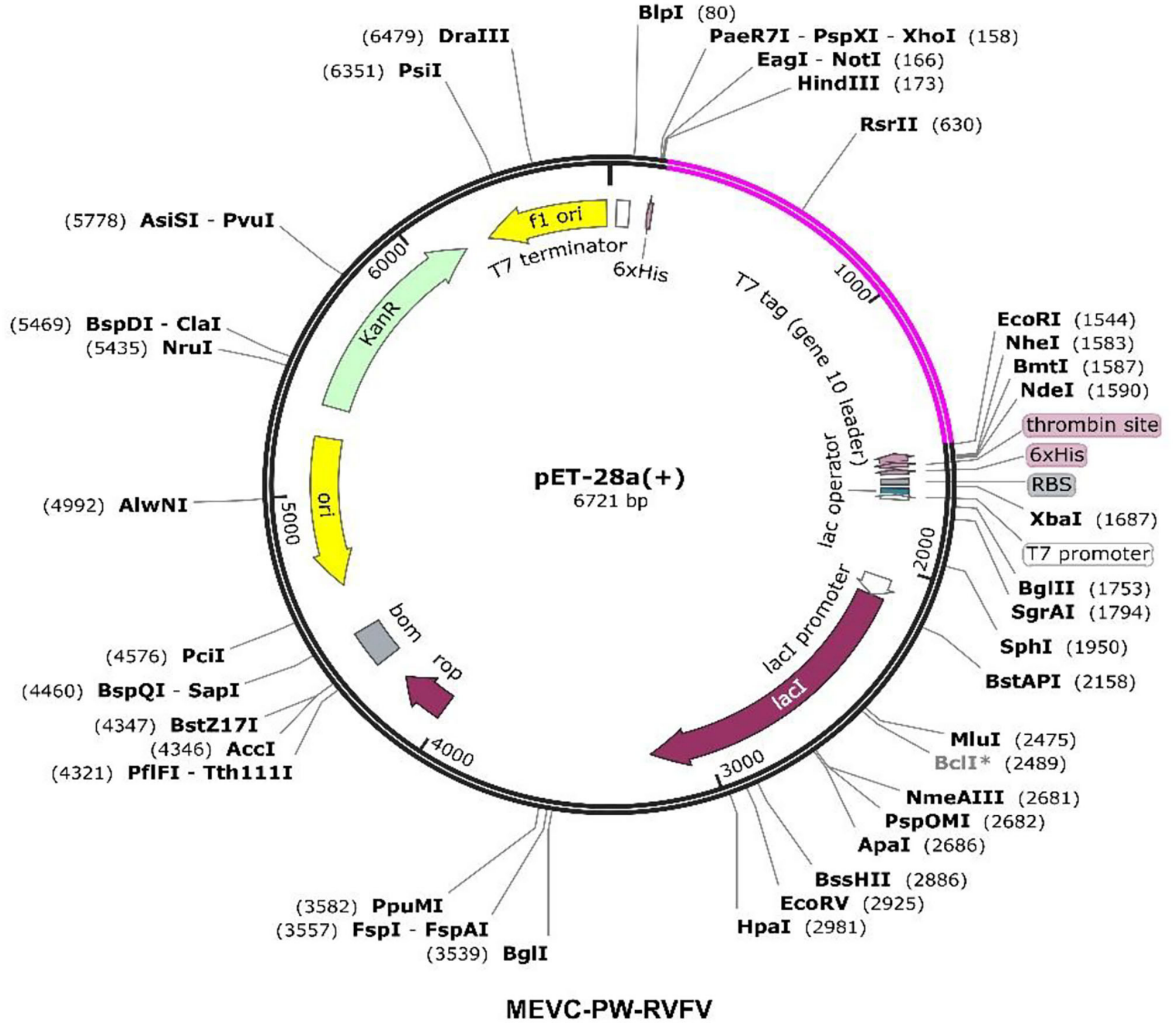
Constructed plasmid maps with pink-colored inserts for the designed MEVC against the whole proteome of RVFV.

It is important for a reverse-translated vaccine construct to demonstrate protein expression in the *E. coli* system.This is vital to acquire the optimized DNA sequence and insert it properly into the *E. coli* expression vector before proceeding with further experimental designs (Naz et al., 2021). A construct without any inserted vaccine construct served as a negative control in this analysis. Using the *in-silico* modeling approaches, we designed the corresponding clones for each of the MEVC constructs. This was achieved through codon optimization, followed by confirmation of putative expression in the *E. coli* (K-12) strain represented by a CAI value for each construct (Table 7). The optimized sequence was then added to a pet28a (+) vector with the utility of SnapGene. The specific sites of restriction enzymes selected for insertion of the optimized sequence into the vector were EcoR1 and Xho1. The final plasmid maps were obtained for each protein-specific MEVC (Supplementary Figure 6), and the whole proteome-specific vaccine design was constructed as shown in Figure 5.

### Immune Simulation of the MEVCs

**Figure 6:**
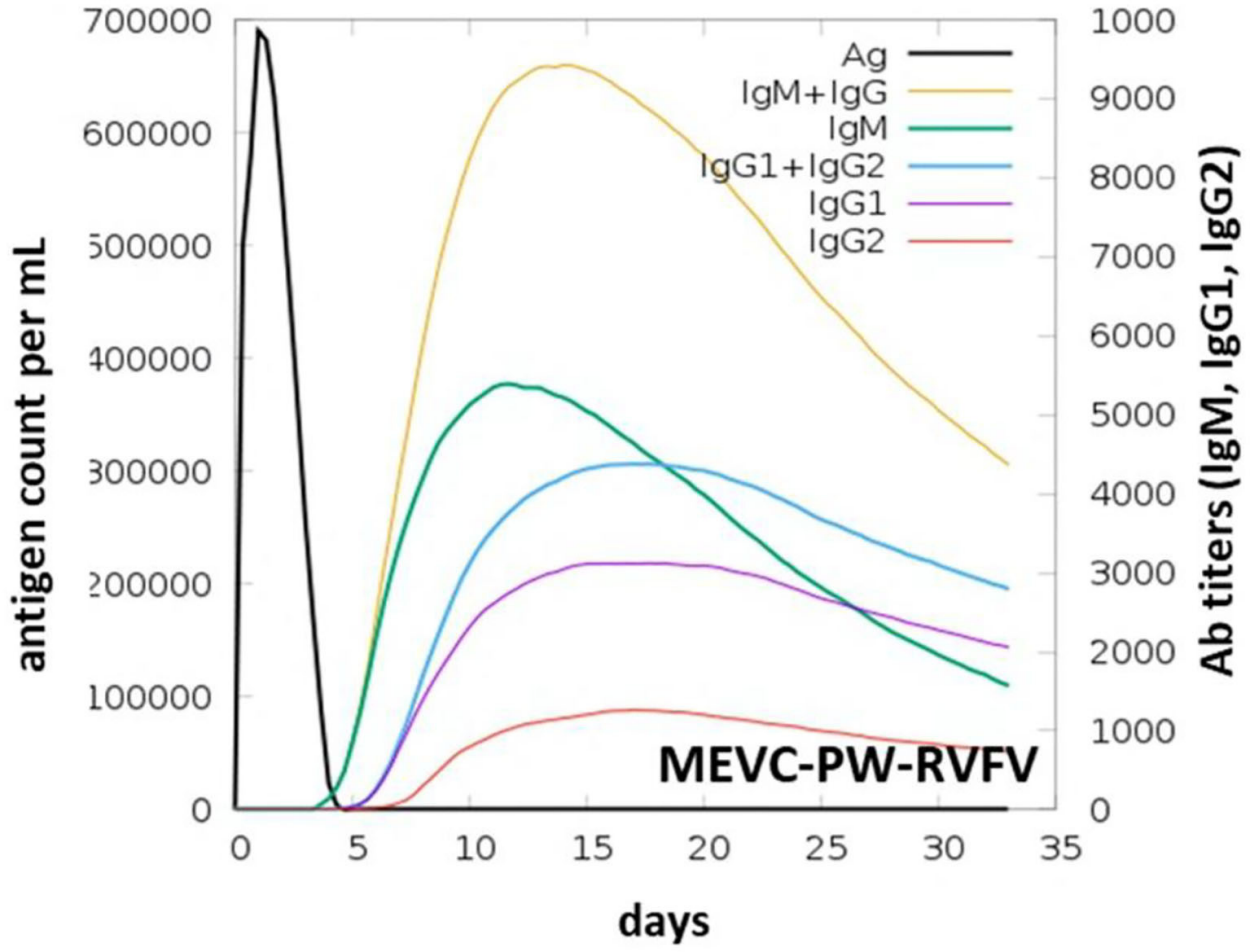
Graph-based representation of induced immune response in the form of Ab titers observed through an immune-simulation graph of MEVC designed against the whole proteome of RVFV.

Using the immune simulation approach, we evaluated the potential immune response induction in the form of predicted antibody titers produced against each MEVC construct. It can be observed that the injected antigens (Supplementary Figures 7A–D) after achieving the highest antigen counts on day 2 were slowly neutralized till day 5. Afterward, a strong antibody (IgM+ IgG) response has been observed with the highest achieved titers of >9,000 xx/ml between 10 and 15 days for MEVC-NP (Supplementary Figure 7A). For other MEVC designs targeted against each protein (MEVC-NP, MEVC-L, and MEVC-NSP), the antibody response was also found to be higher, ranging from 2,500 to 9,000 (Supplementary Figures 7B,D). The significantly higher antibody titers for each MEVC were observed between 10 and 15 days. In addition, the proteome-wide MEVC design against RVFV (“MEVC-PW-RVFV”) also showed the highest response of about >9,000 xx/ml for combined (IgM+ IgG) antibody titers (Figure 6). This was followed by IgM-specific antibody titers of about 5,000 xx/ml. All the obtained graphs depicted the higher immunogenic potential of each designed vaccine to trigger a robust immune response.

This also suggested the potential of designed MEVCs in this study to produce protective immunity against RVFV. However, further demonstrations through lab experiments are required for the confirmation of the efficacy of each MEVC. The immune-simulation graphs obtained for negative control (Supplementary Figure 8) and each of the designed MEVCs are shown in Supplementary Figure 5.

### Molecular Docking Analysis

**Figure 7:**
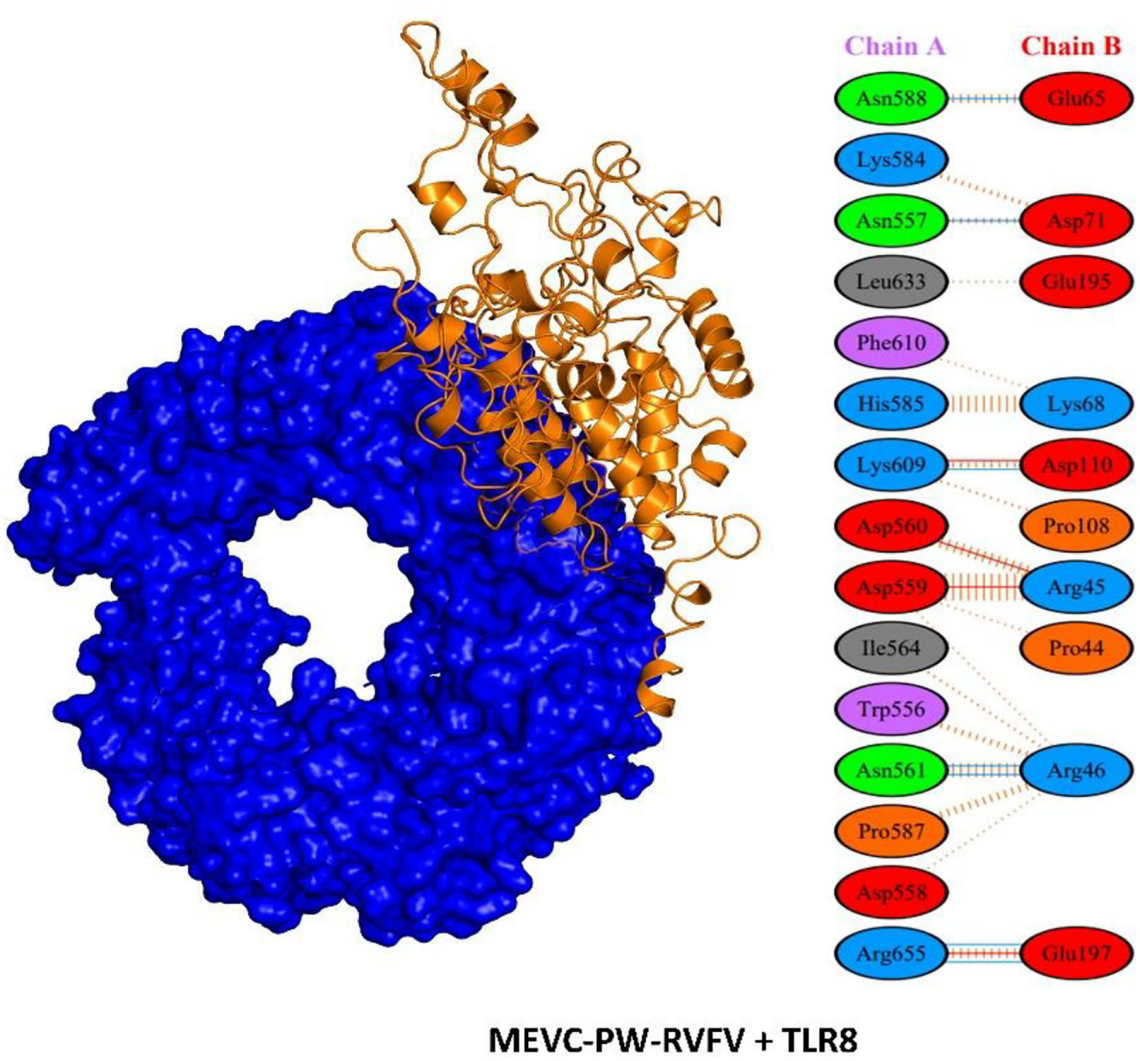
Structural representation of docking complex of MEVC and interaction pattern of the MEVC designed against the whole proteome of RVFV with human TLR8.

It is important to evaluate the potential interaction of the vaccine designs with the target immune receptors to confirm the generation of a stable immune response (Aslam et al., 2021; Ullah et al., 2021). This evaluation and characterization of potential interactions were performed through molecular docking analysis of each MEVC with Toll-like receptor 8 (TLR8) by using PDB-ID “6KYA” (Jiang et al., 2020). Such, Toll-like receptors (TLRs) play a crucial role in the activation of innate immunity by detecting conserved pathogen-associated molecular patterns (PAMPs) and production of adaptive immune response against the invading pathogens (Fatima et al., 2021; Ullah et al., 2021). Specifically, TLR8 has been widely implicated in the recognition of RVFV (McElroy and Nichol, 2012; Hise et al., 2015) and induction of immune response. The docking complex of the negative control docked with TLR8 is shown in Supplementary Figure 8A. Although the negative control showed few interactions with the human TLR8 (Supplementary Figure 8B), the loops and unfolded docked structure of the negative control showed invalid results. The docking complexes for each of the designed MEVCs and TLR8 with proper folding are shown in Figure 7.

**Figure 8:**
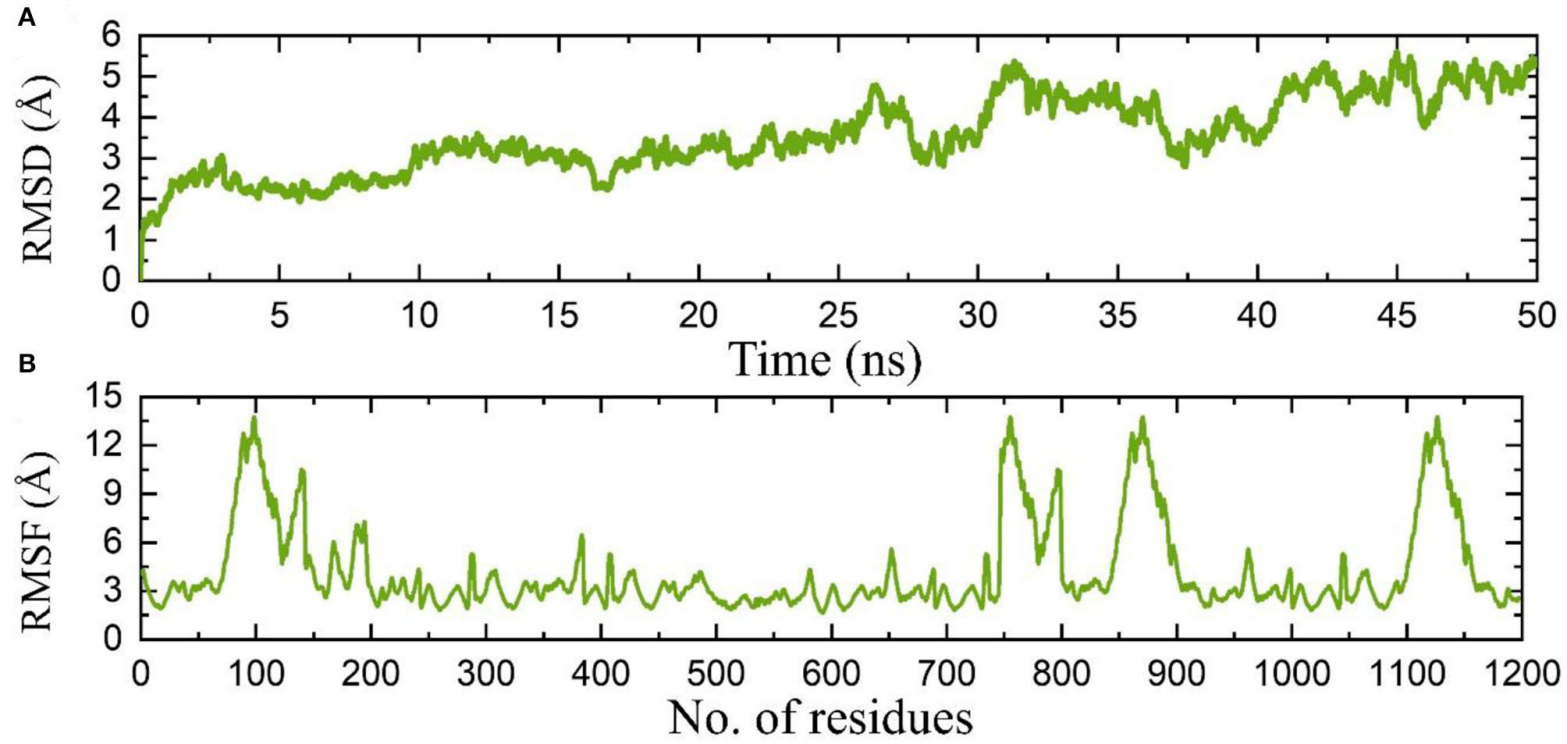
**(A)** Root mean square deviation based on stability of the complex, while **(B)** shows the RMSF of the complex.

Molecular dynamics simulation of the proteome-wide vaccine construct demonstrated stable dynamics with no significant structural perturbation. An average RMSD for the complex was calculated to be 8.0 Å, which is convincible for the space occupied by such large atoms. On the other hand, the RMSF also demonstrated similar flexibility, and regions 50–150, 740–800, 830–900, and 1,090–1,150 demonstrated higher fluctuations where loops are distributed. Consequently, this shows that the binding of the proteome-wide vaccine construct is stable validated by the MD results. The RMSD and RMSF results are given in Figures 8A,**B**, respectively.

Furthermore, PDBePISA (Paxman and Heras, 2017) was used to determine the interaction patterns between the MEVCs and human TLR8 (Krissinel and Henrick, 2007). This analysis revealed a binding score ranging from −45 to −60. Among these constructs, the highest binding score was observed for the MEVC-NSP + TLR8 complex (Supplementary Figure 9C). The details of the binding scores and interaction patterns, including the number of hydrogen bonds and salt bridges for all the docking complexes, are given in Table 8. The highest number of hydrogen bond (7) contacts were found for MEVC-PW + TLR8 docking complex (Figure 7). Similarly, the number of salt bridges (4) was also found to be highest for MEVC-PW + TLR8 docking complex. Overall, the interacting patterns for all the docking complexes (Supplementary Figure 9) suggested a higher binding affinity of the designed MEVCs for the human TLR8. The MEVC-NP + TLR8 complex reported the interactions between these residues: Thr45-Pro40, His46-Pro40, and GLU50-ARG46. MEVC-L + TLR8 formed interactions between ARG551-TYR89, ARG582-TYR89, ARG582-GLU95, ARG501-GLU97, ARG582-GLU95, and LYS631-GLU109. MEVC-NSP + TLR8 established different contacts between GLN451-ASN127, VAL452-ARG151, SER454-TRP129, THR456-ARG126, GLU510-GLY102, and Glu457-Arg151. MEVC-GP + TLR8 reported interactions between ASN431-LYS134, THR456-PRO103, GLU457-LYS134, GLU434-LYS131, and GLU484-ARG104. MEVC-PW + TLR8 revealed interactions between ASN557-ASP71, ASN561-ARG46, ASN588-GLU65, LYS609-ASP110, ARG655-GLU197, ASP559-ARG45, ASP560-ARG45, LYS609-ASP110, and ARG655-GLU197. The interaction patterns followed in all the docking complexes for each vaccine construct are shown in Supplementary Figure 10.

**Table 8:** Details of binding scores and identified interaction patterns between the different MEVCS and human TLR8.

**Docking complex (Vaccine name + TLR8)**	**Binding energy scores**	**Number of hydrogen bonds**	**Number of salt bridges**
MEVC-NP + TLR8	−45.93	4	2
MEVC-L + TLR8	−50.31	4	3
MEVC-NSP + TLR8	−60.87	5	1
MEVC-GP + TLR8	−56.32	3	2
MEVC-PW + TLR8	−57.6	7	4
MEVC-NEG + TLR8	−32.46	2	1

### Limitations of the Study

The study demands additional experimental validation of the final vaccine designs. However, several physicochemical properties explored for each vaccine design require verification in the wet lab. Similarly, the feasibility of expression and purification, as confirmed through *in-silico* approaches, requires further testing through the use of recombinant DNA technologies.

## Conclusion

Till date, there is no available licensed vaccine against the RVF virus for human use. In this study, the immune-informatics approach has been utilized to shortlist putative immune epitopes for each target protein of RVFV and used in the final construction of MEVCs. Several highly antigenic immune epitopes, including T cell, B cell, and HTL, were mapped for each of the four target proteins (NP, L, NSP, and GP) of the RVF virus. Moreover, a proteome-wide multi-epitope-based vaccine (MEVC-PW-RVFV) construct has also been proposed. All the constructed MEVCs have been 3D modeled and evaluated for potential immunization. The present study provides new insights into the development of future vaccine development against the emerging virus. The characterization of epitopes and whole proteome-specific MEVC design with evaluated high immunogenic potential suggests experimental processing of these vaccine designs against the Rift Valley fever virus. However, further experiments may validate the immune reinforcement potential of these final vaccine candidates.

## Data Availability Statement

The original contributions presented in the study are included in the article/Supplementary Material, further inquiries can be directed to the corresponding authors.

## Author Contributions

AA designed, performed, and write this study.

## Conflict of Interest

The author declares that the research was conducted in the absence of any commercial or financial relationships that could be construed as a potential conflict of interest.

## Publisher's Note

All claims expressed in this article are solely those of the authors and do not necessarily represent those of their affiliated organizations, or those of the publisher, the editors and the reviewers. Any product that may be evaluated in this article, or claim that may be made by its manufacturer, is not guaranteed or endorsed by the publisher.
