# Supplementary material for: Proteome-Wide and Protein-Specific Multi-Epitope Vaccine Constructs Against the Rift Valley Fever Virus Outbreak Using Integrated Omics Approaches
Source: Front Microbiol. 2022 May 31;13:921683. doi: 10.3389/fmicb.2022.921683 (PMC9195176; doi:10.3389/fmicb.2022.921683)
Supplement: Supplementary file 1 [file Table_1.DOCX]

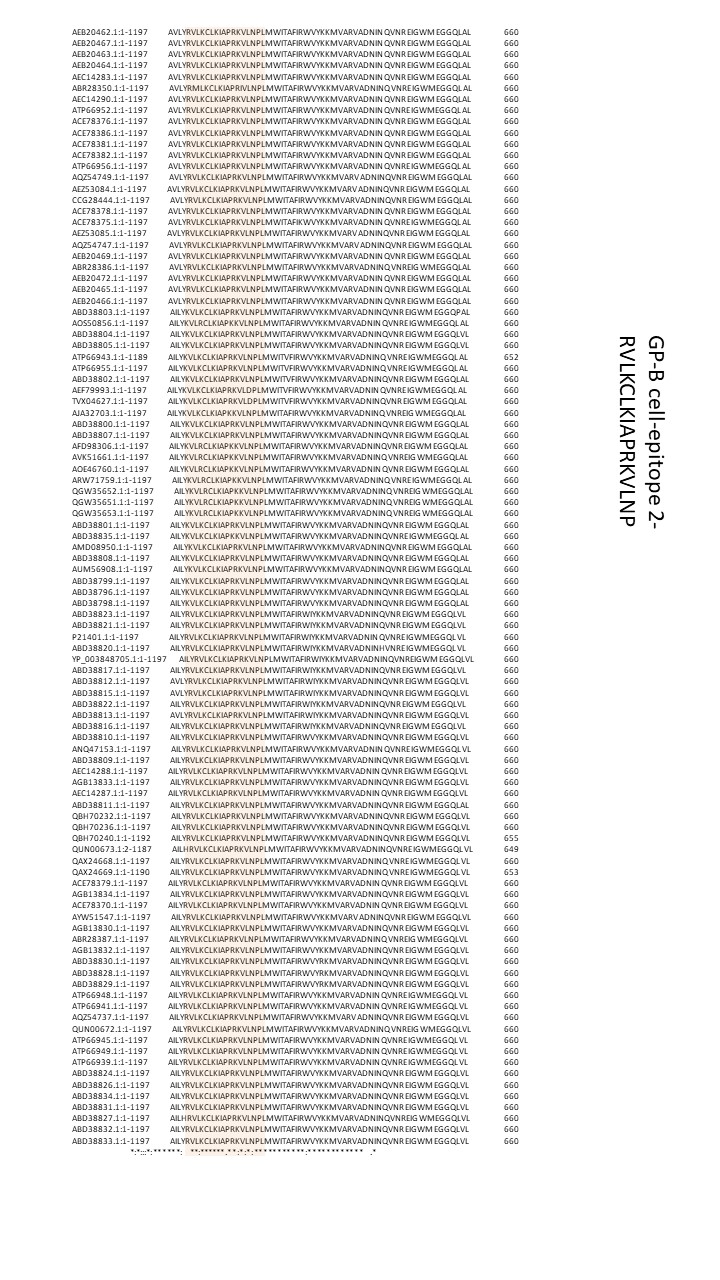

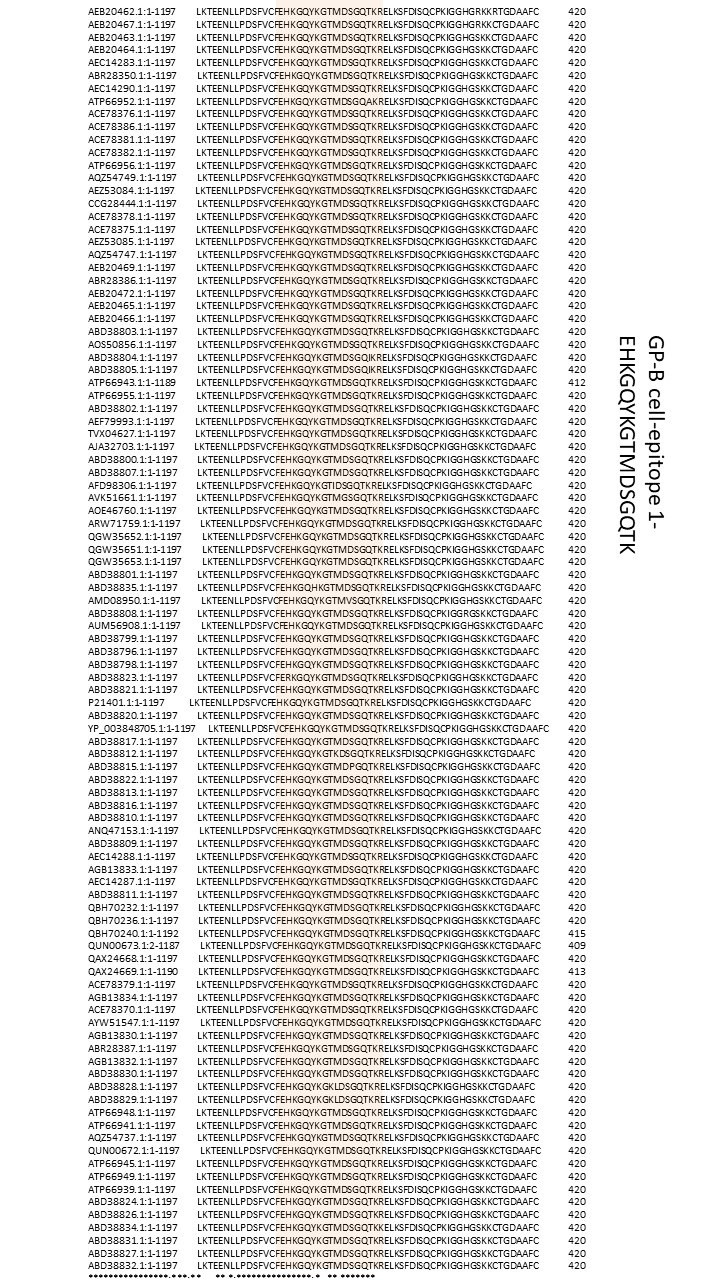


**B**

**A**

**Figure S1 (A-B): Showing the different epitopes conservancy of analyzed B cell epitopes for Glycoprotein (GP).**

**C**


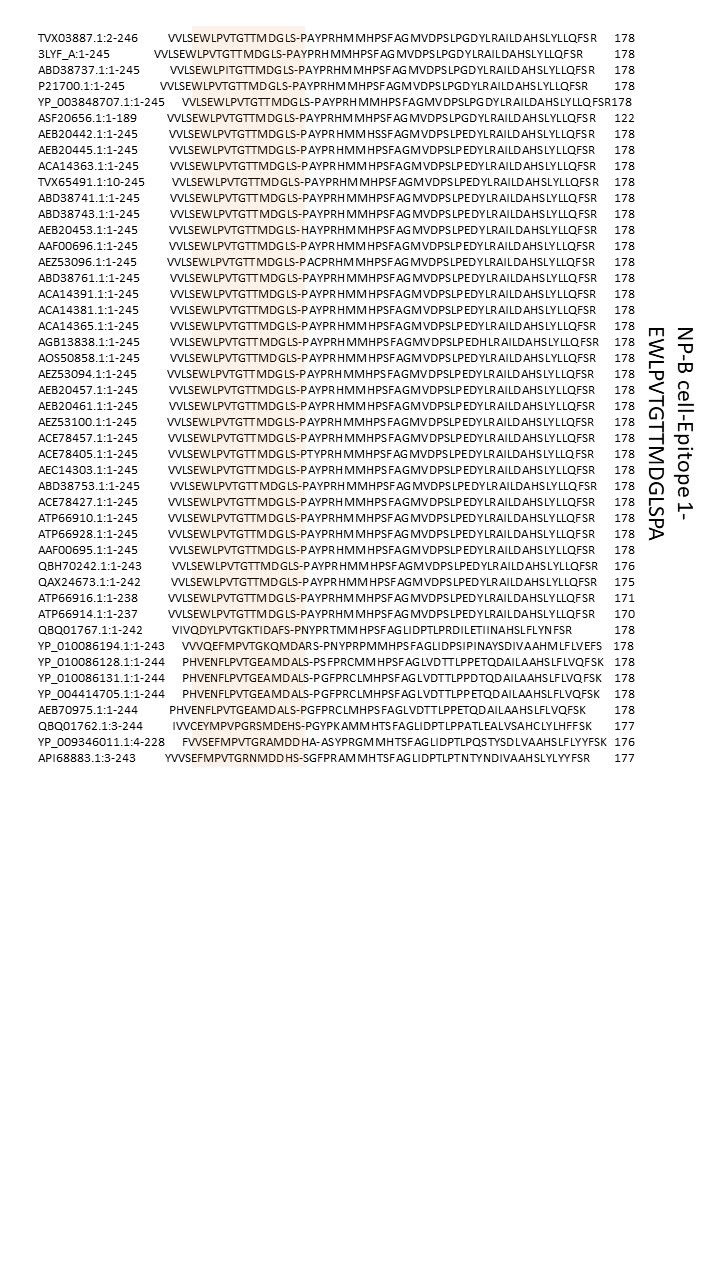


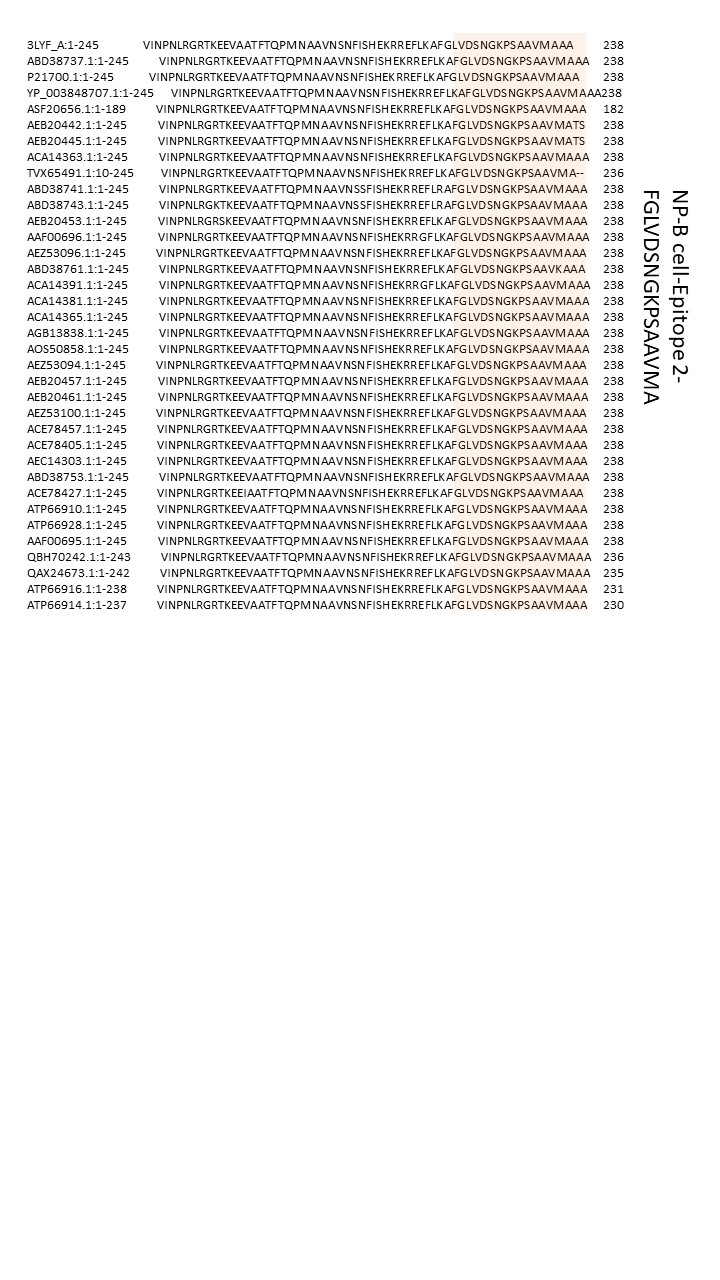


**D**

**Figure S1 (C-D): Showing the different epitopes conservancy of analyzed B cell epitopes for Nucleoprotein (NP).**


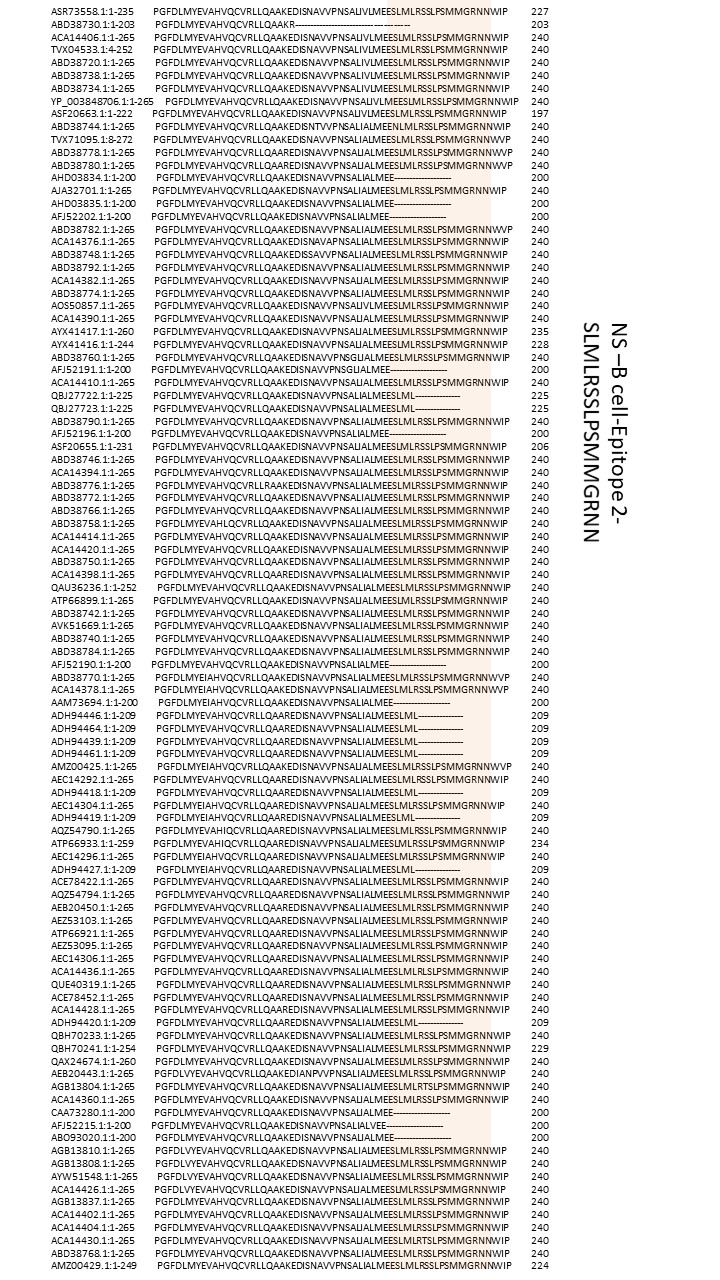

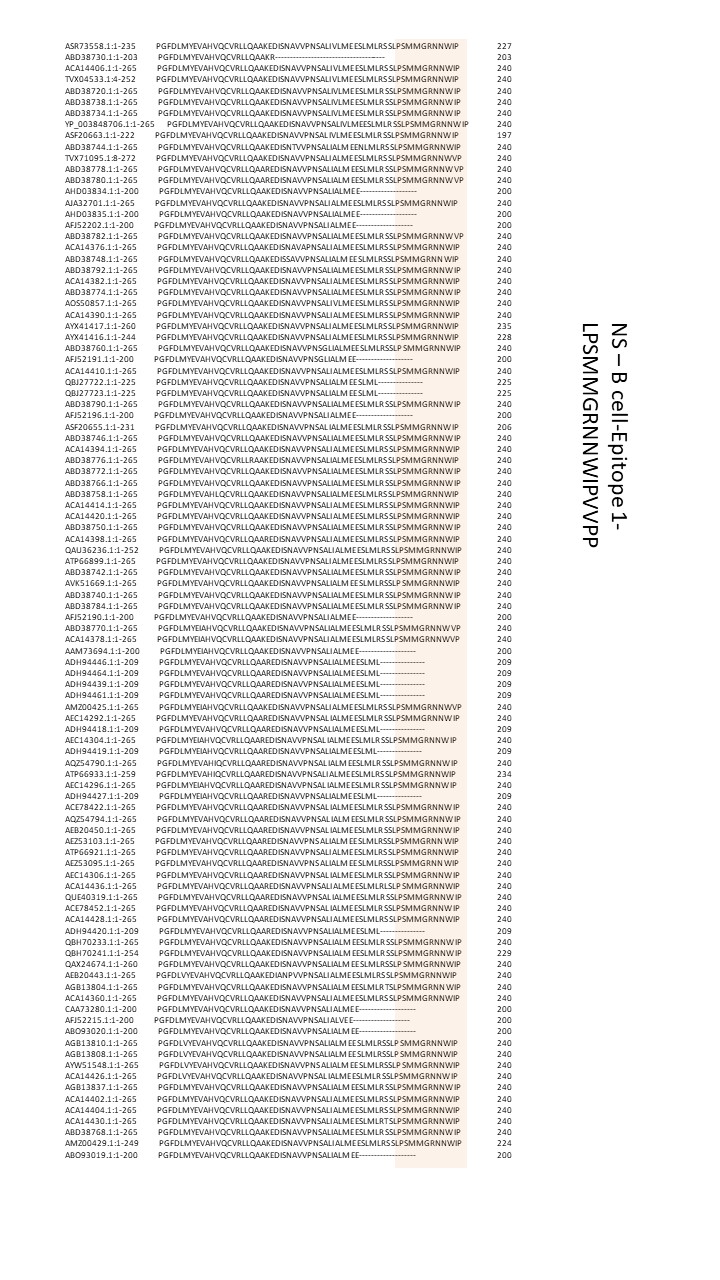


**F**

**E**

**Figure S1 (E-F): Showing the different epitopes conservancy of analyzed B cell epitopes for Non-structural protein (NSP).**

**
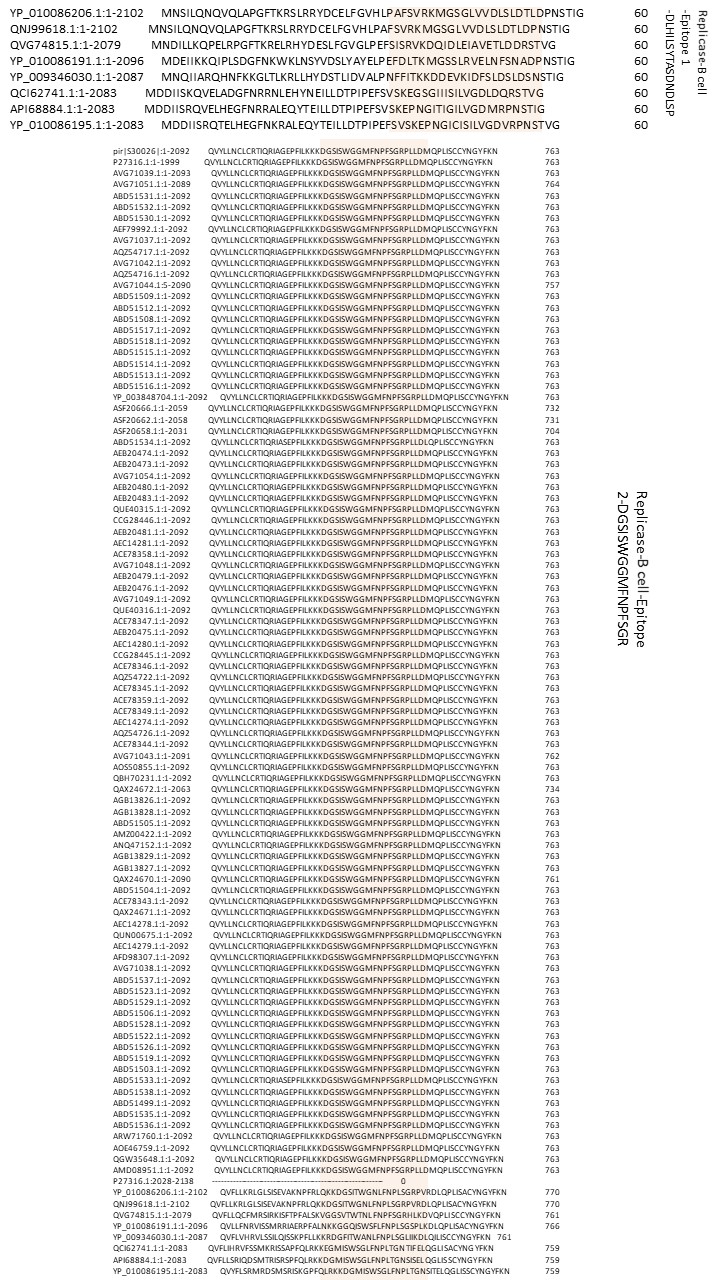
**

**G**

**H**

**Figure S1 (G-H): Showing the different epitopes conservancy of analyzed B cell epitopes for Replicase protein.**


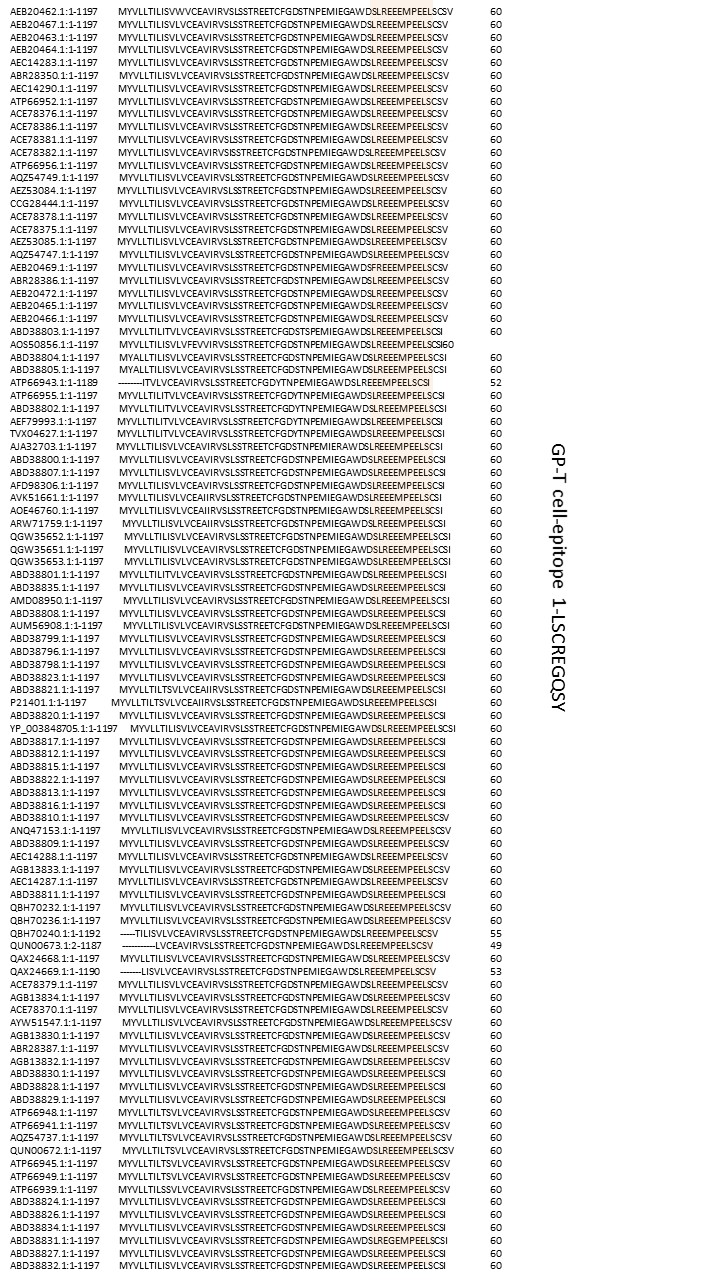

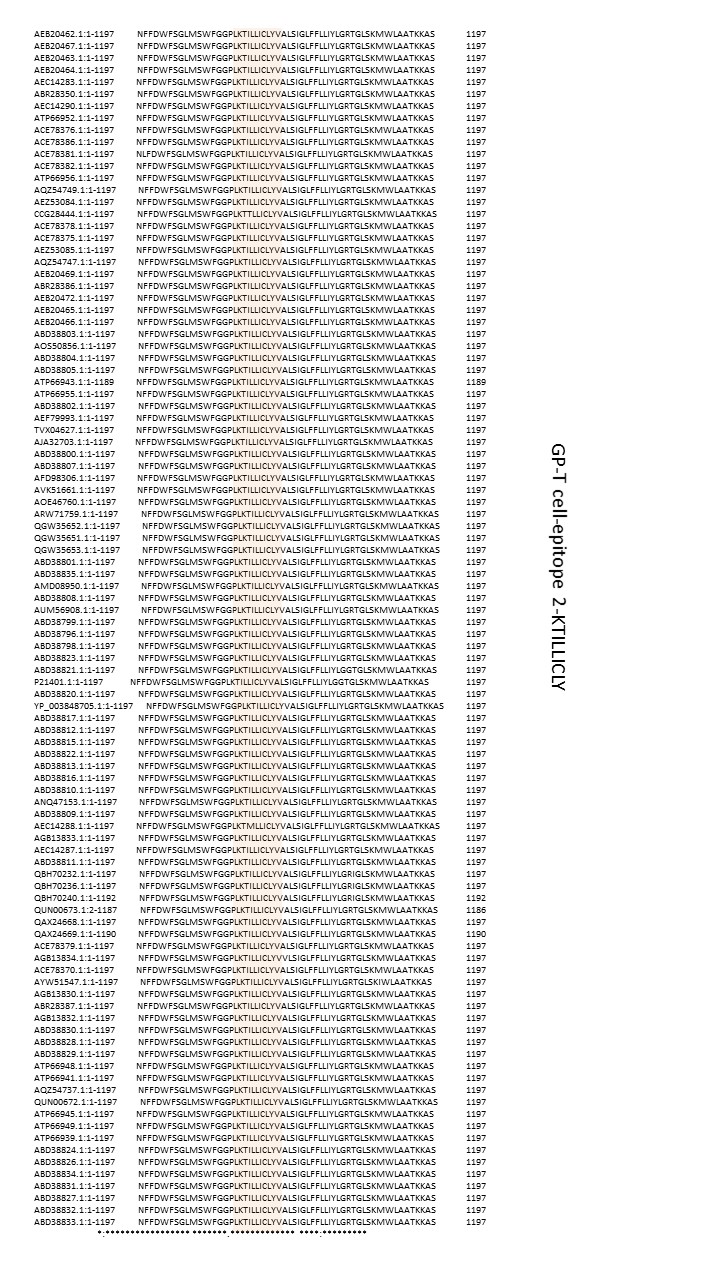


**I**

**J**

**Figure S1 (I-J): Showing the different epitopes conservancy of analyzed T cell epitopes for Glycoprotein (GP).**


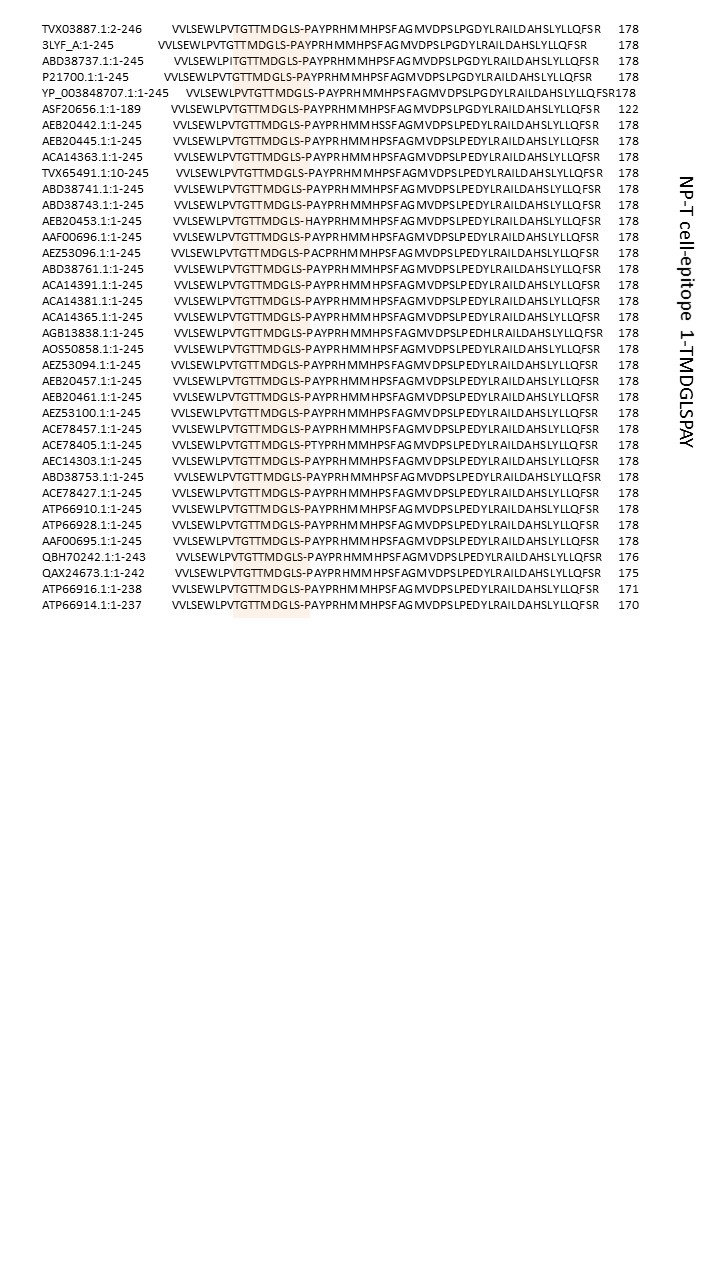


**K**


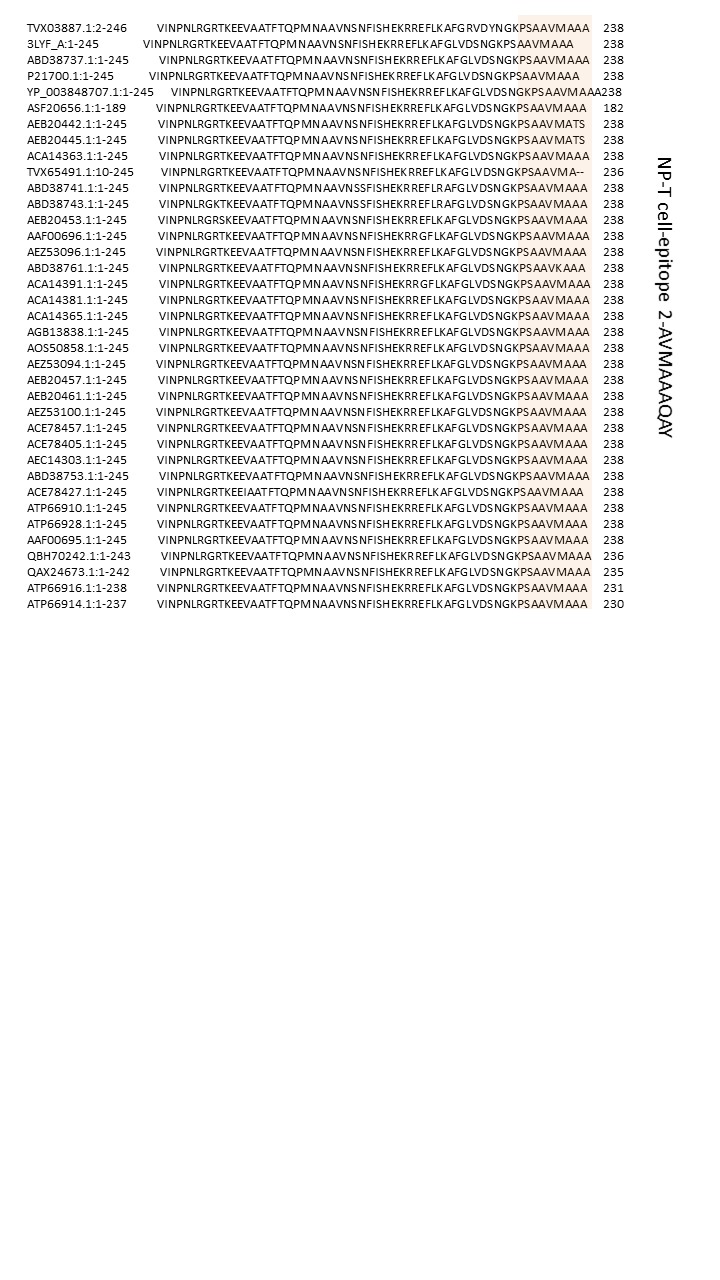


**L**

**Figure S1 (K-L): Showing the different epitopes conservancy of analyzed T cell epitopes for Nucleoprotein (NP).**


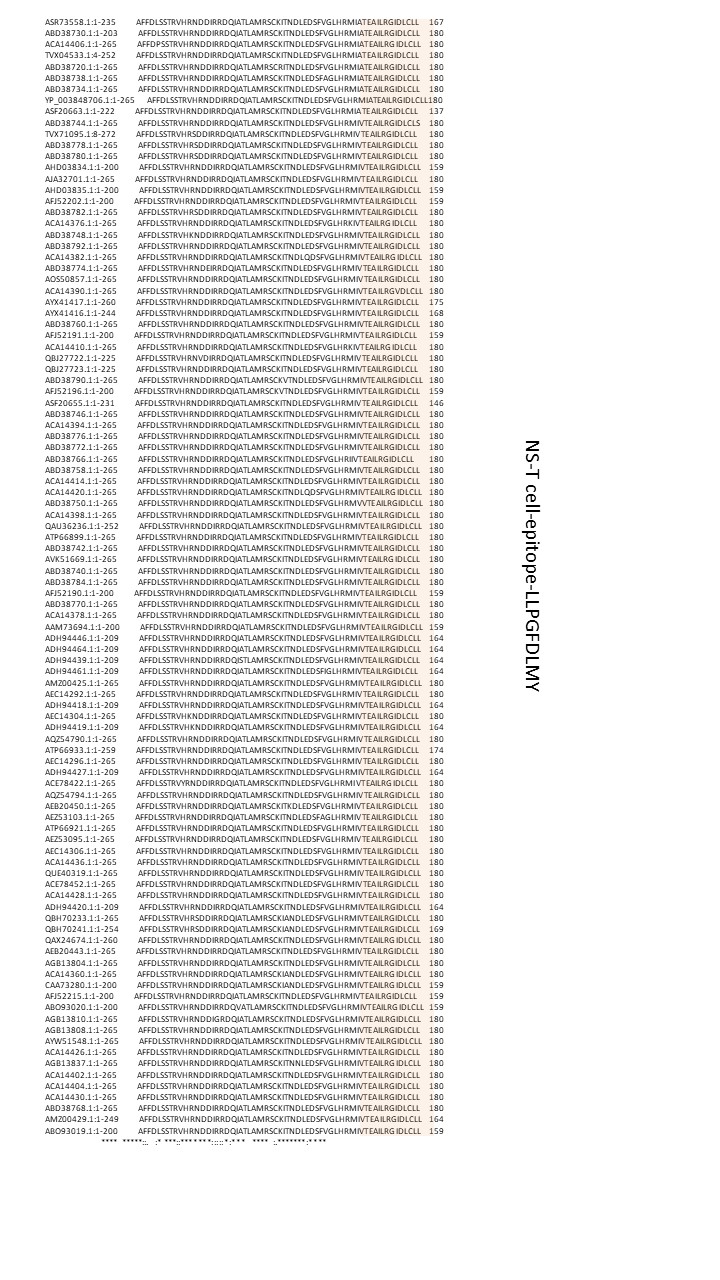


**M**

**Figure S1 (M): Showing the epitope conservancy of analyzed T cell epitope for Non-structural protein (NSP).**


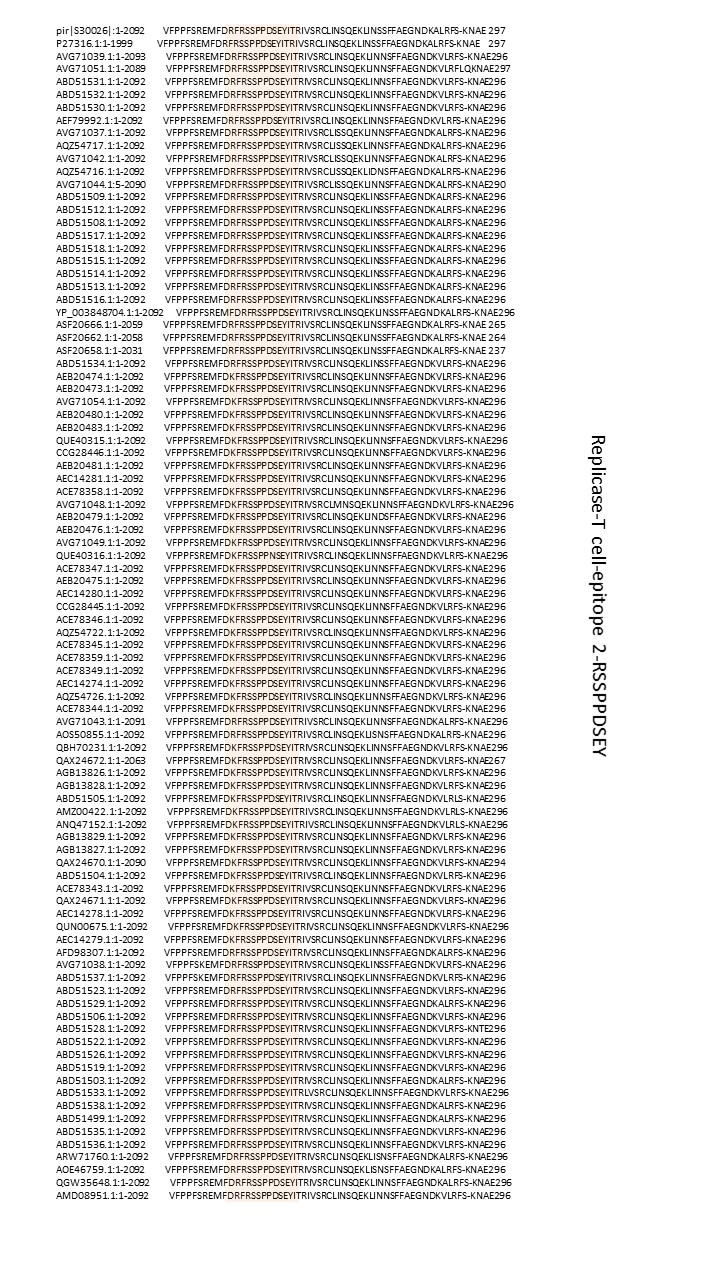

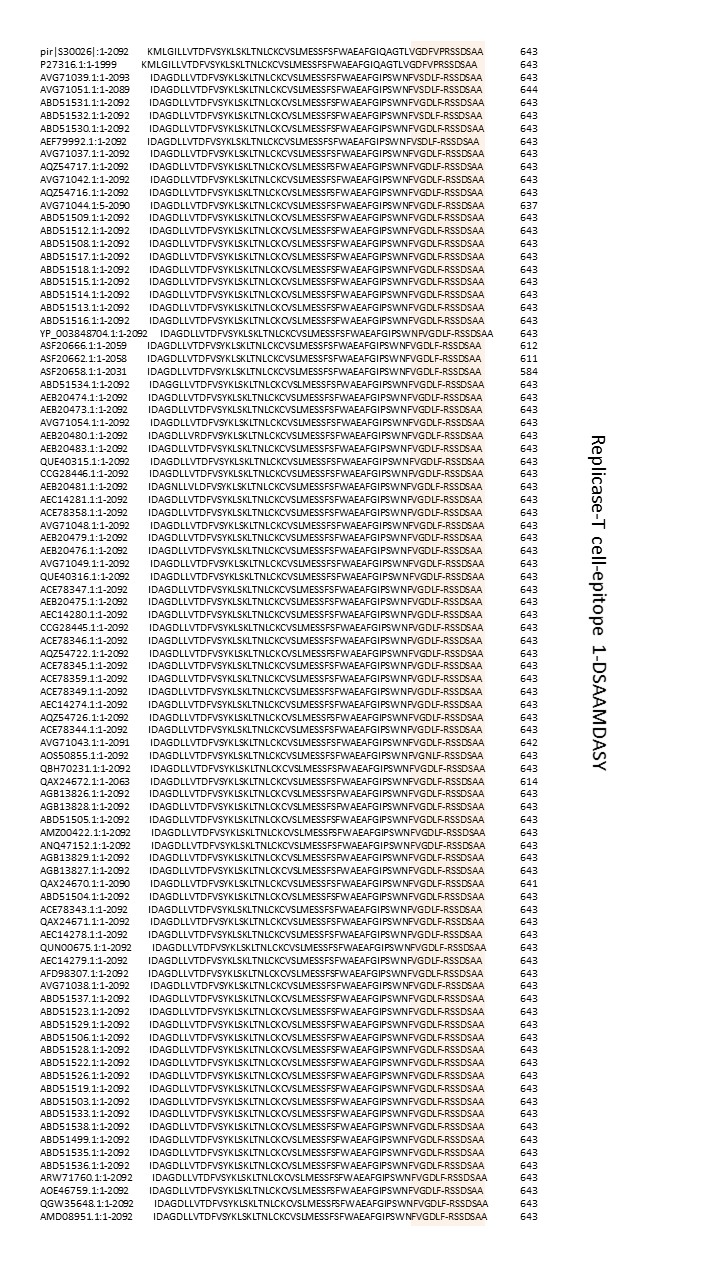


**O**

**N**

**Figure S1 (N-O): Showing the different epitopes conservancy of analyzed T cell epitopes for Replicase.**


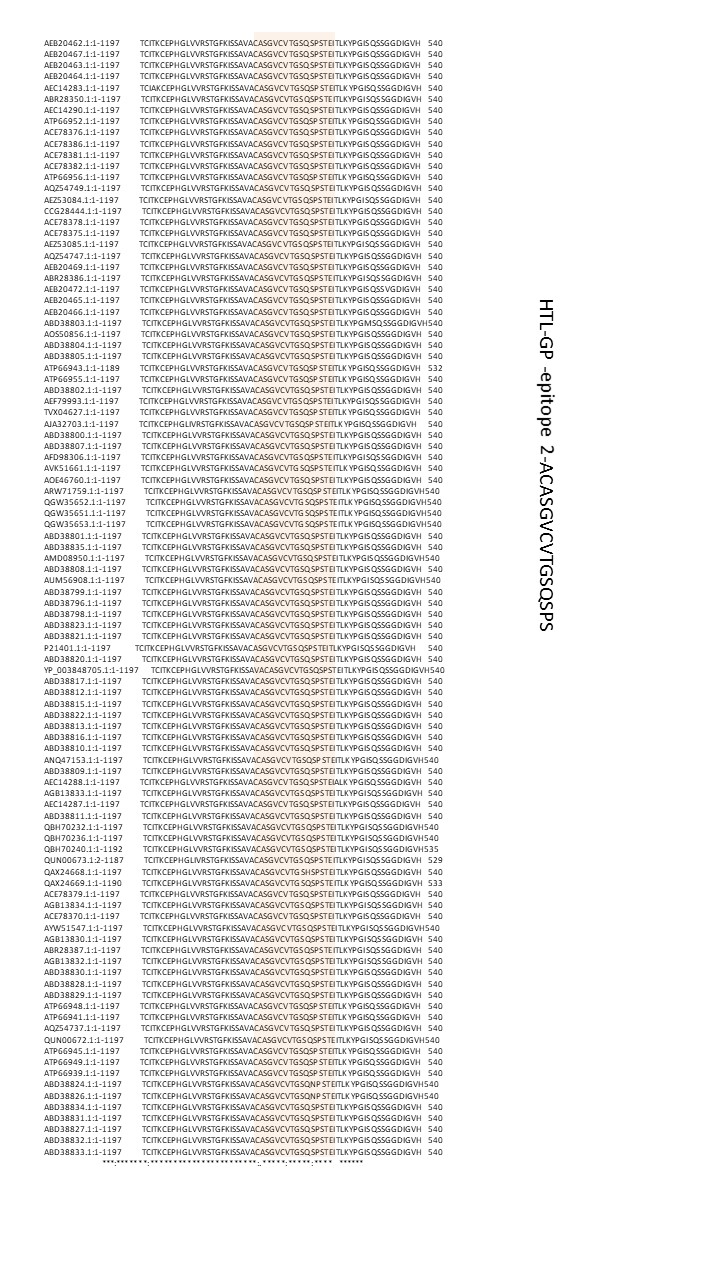

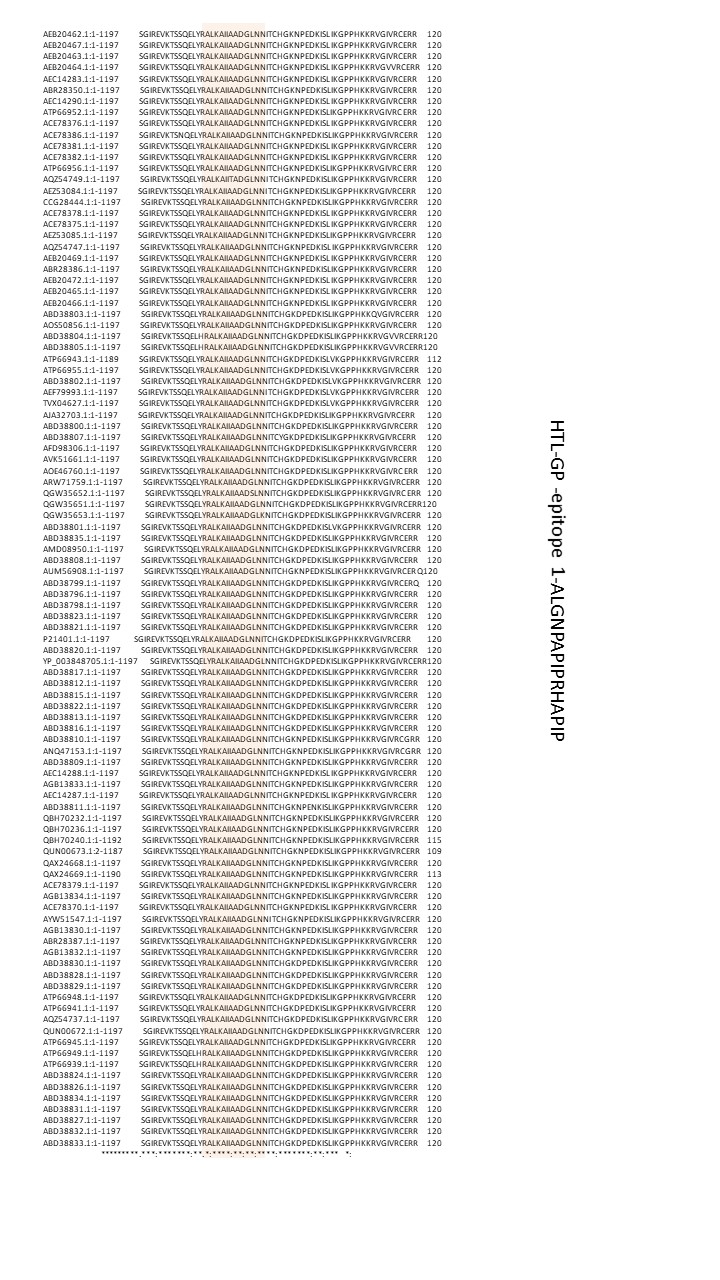


**Q**

**P**

**Figure S1 (P-Q): Showing the different epitopes conservancy of analyzed HTL epitopes for Glycoprotein (GP).**


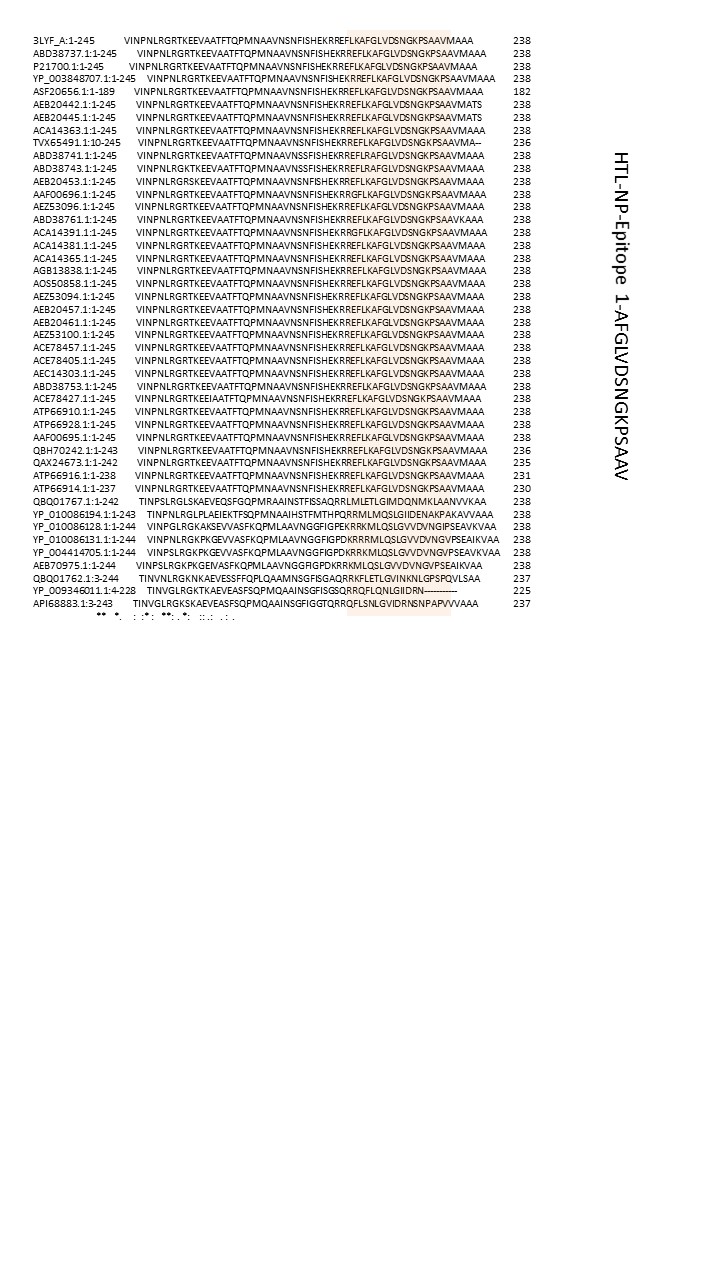

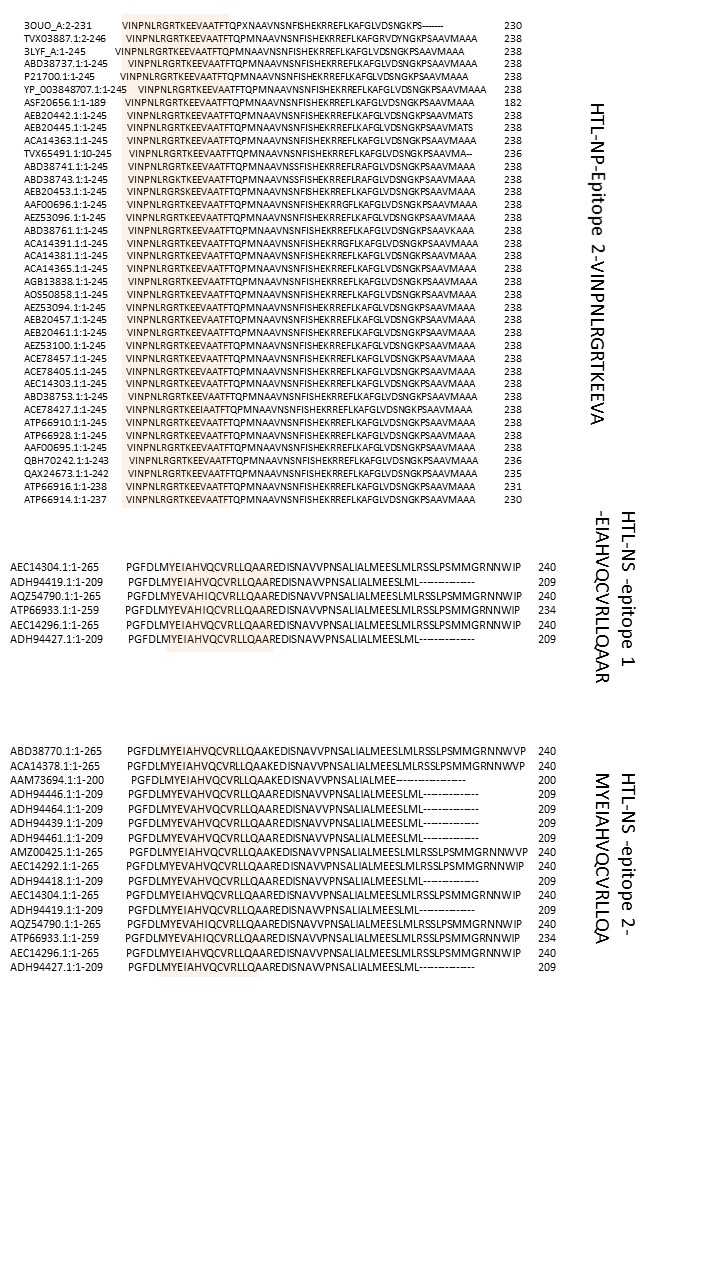


**S**

**R**

**Figure S1 (R-S): Showing the different epitopes conservancy of analyzed HTL epitopes for Nucleoprotein (NP).**


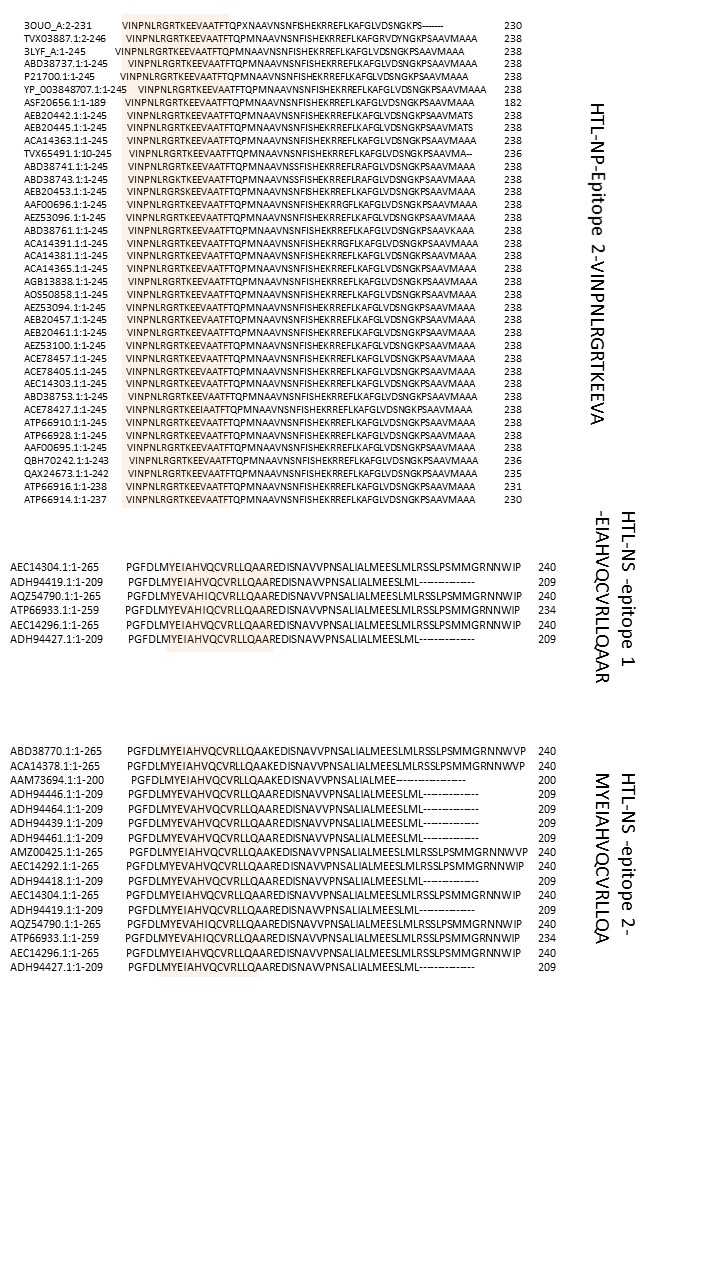


**T**

**U**

**Figure S1 (T-U): Showing the different epitopes conservancy of analyzed HTL epitopes for Non-structural protein (NSP).**


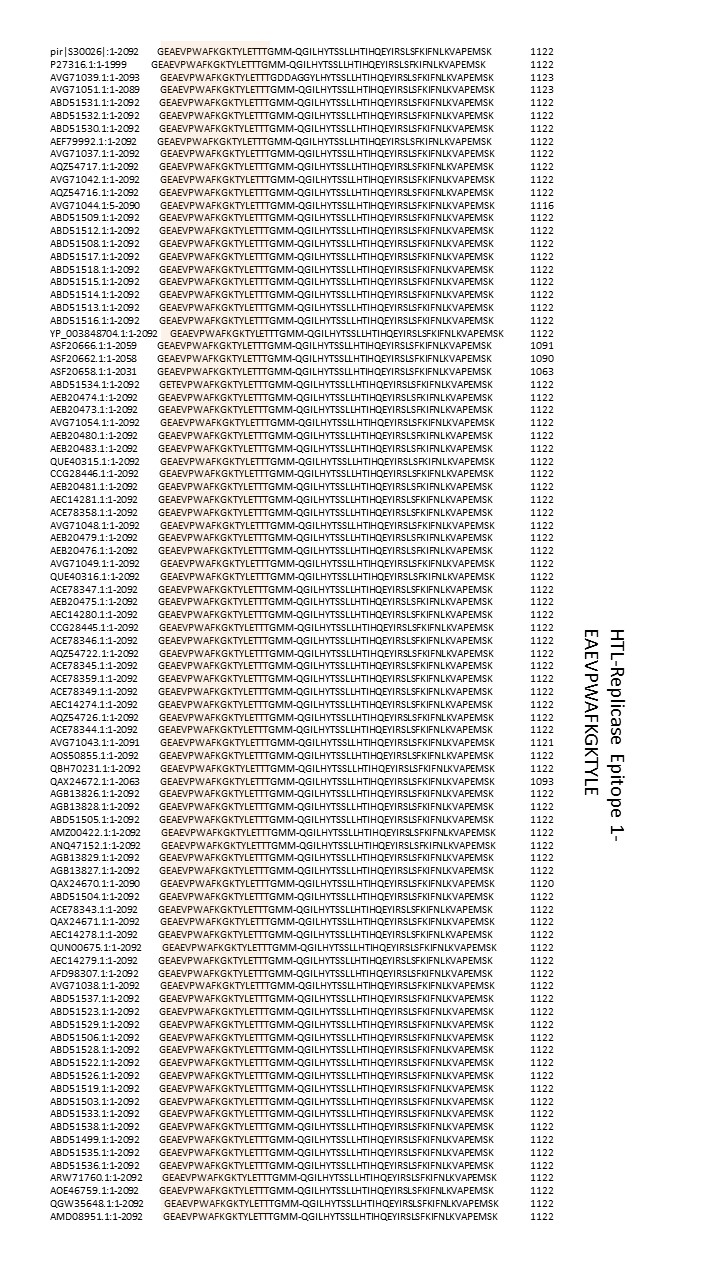


**V**

**W**


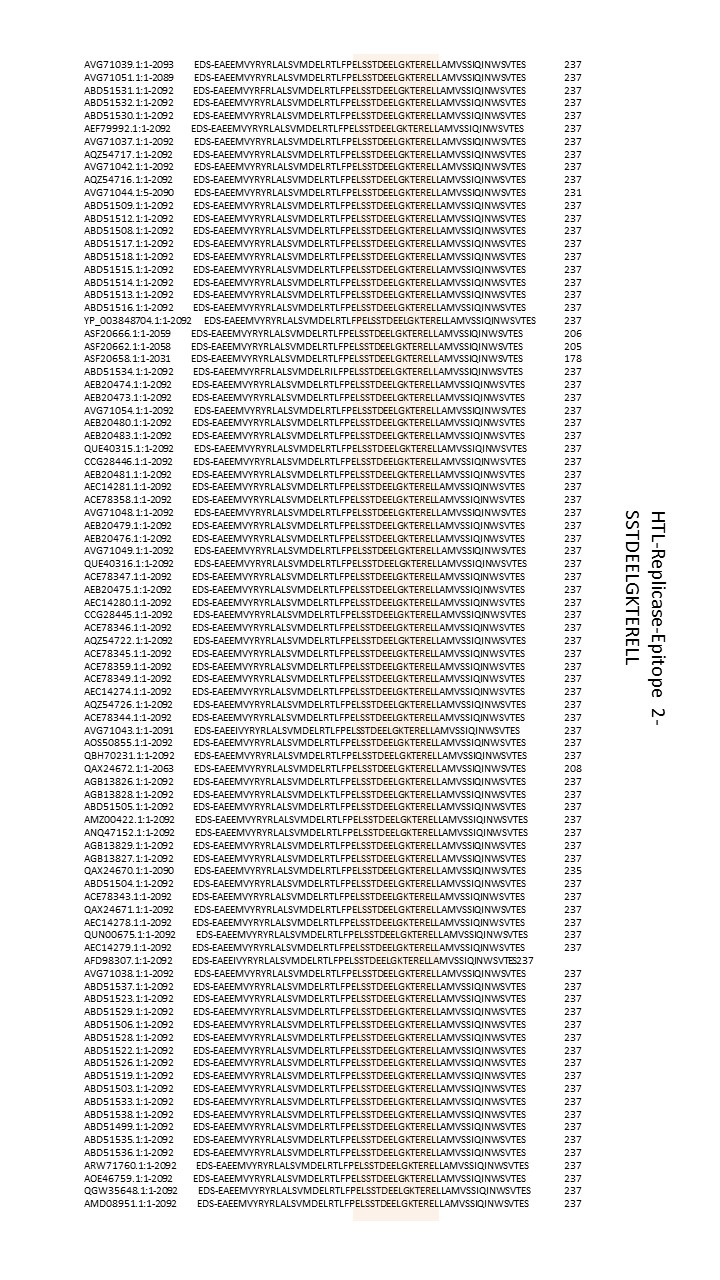


**Figure S1 (V-W): Showing the different epitopes conservancy of analyzed HTL epitopes for Replicase.**


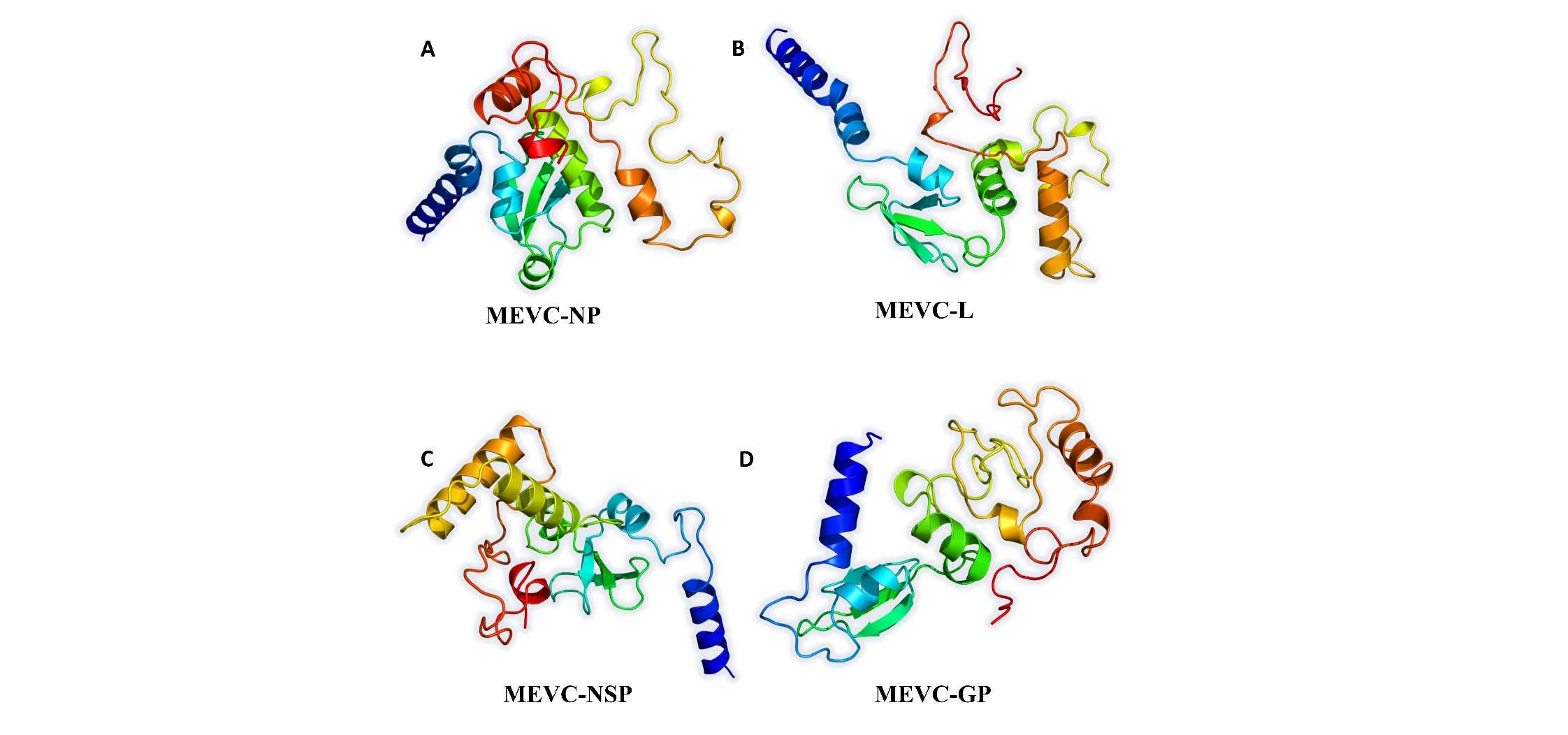


**Figure S2:** Showing the 3D structure models of each protein specific MEVCs. **(A-D)** represents the MEVC designed against each target protein of RVFV i.e., NP, L, NSP and GP.


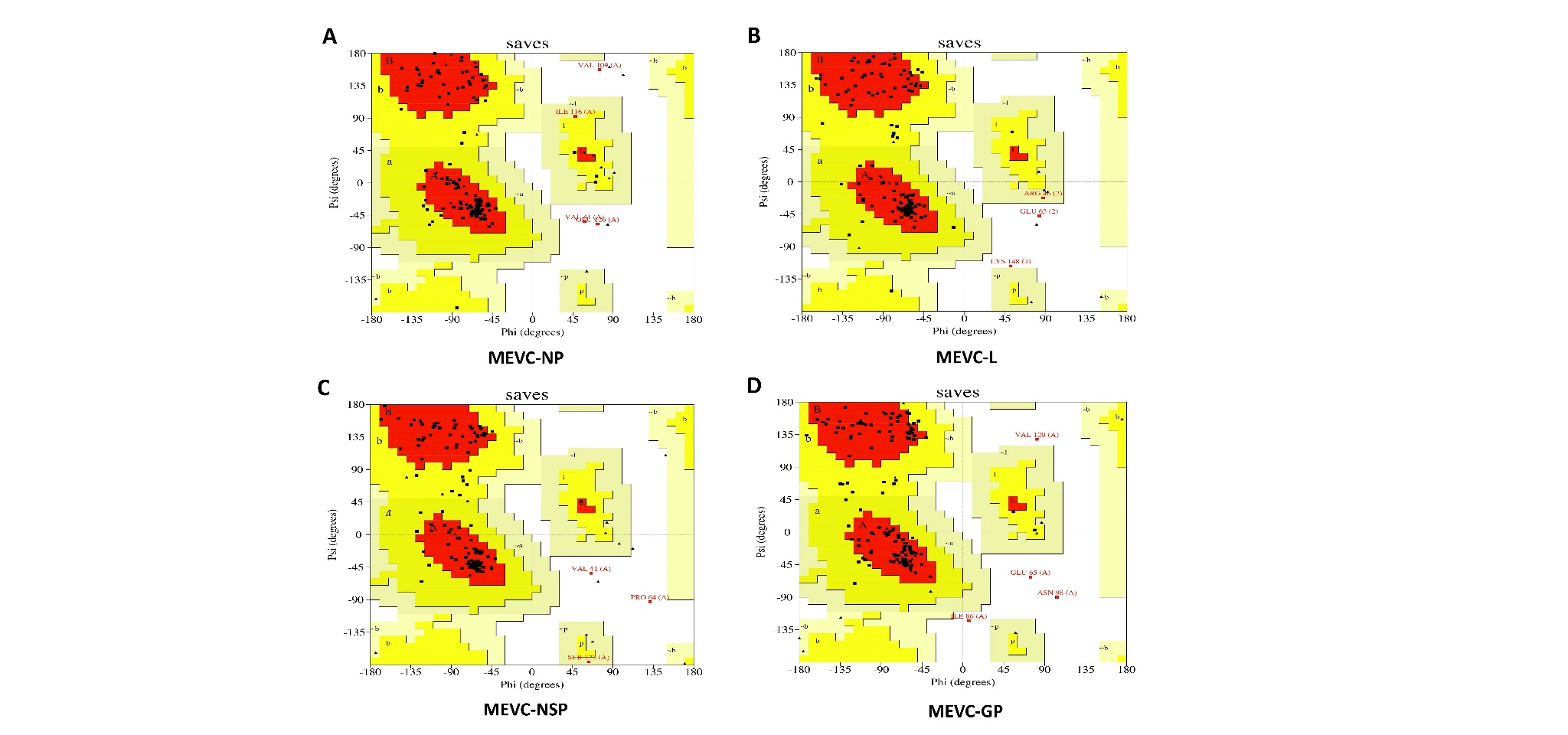


**Figure S3:** Showing structural validation of each MEVC designed against RVFV through Ramachandran plot. **(A-D)** represents the Ramachandran plot of MEVCs designed against each target protein i.e., NP, L, NSP and GP, respectively.

**
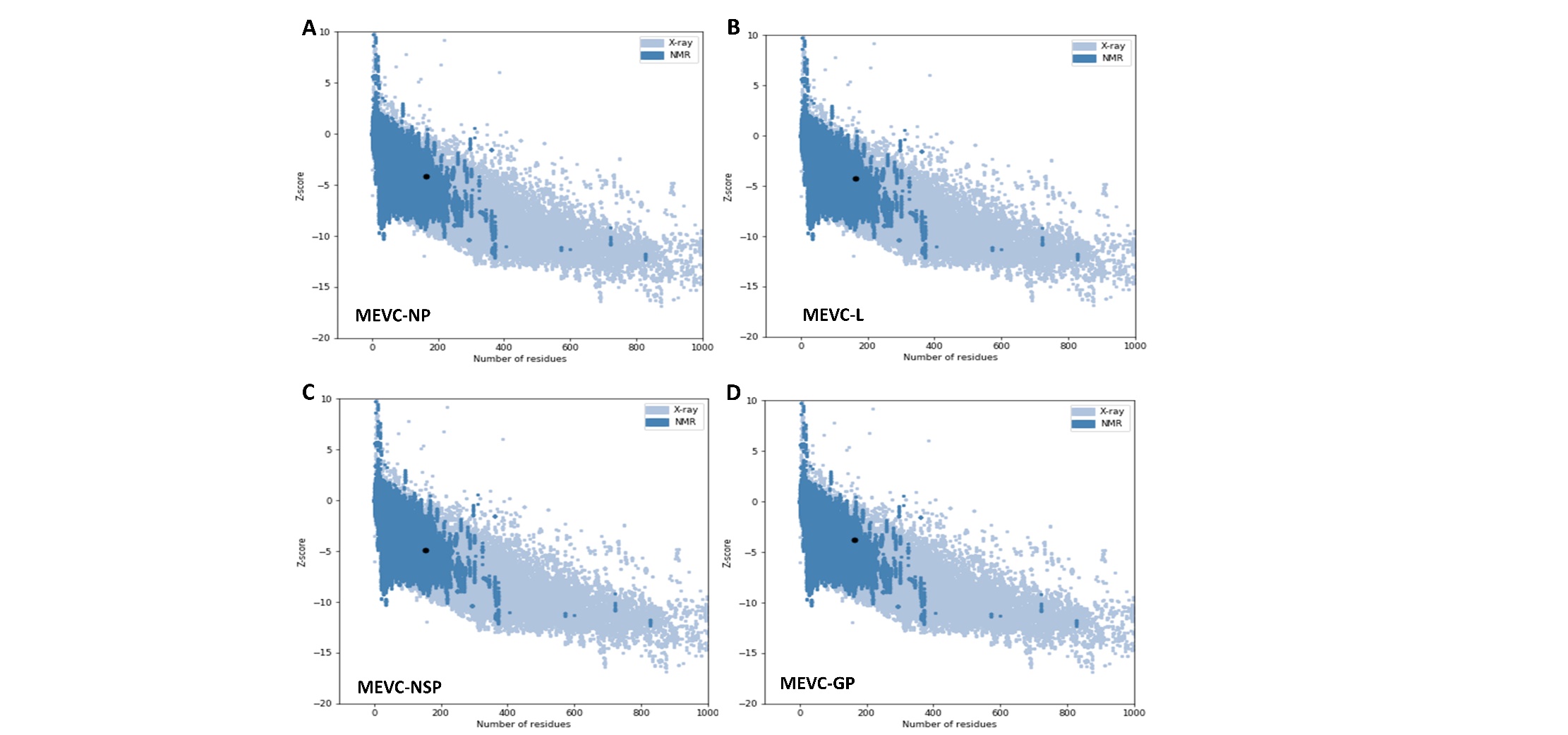
**


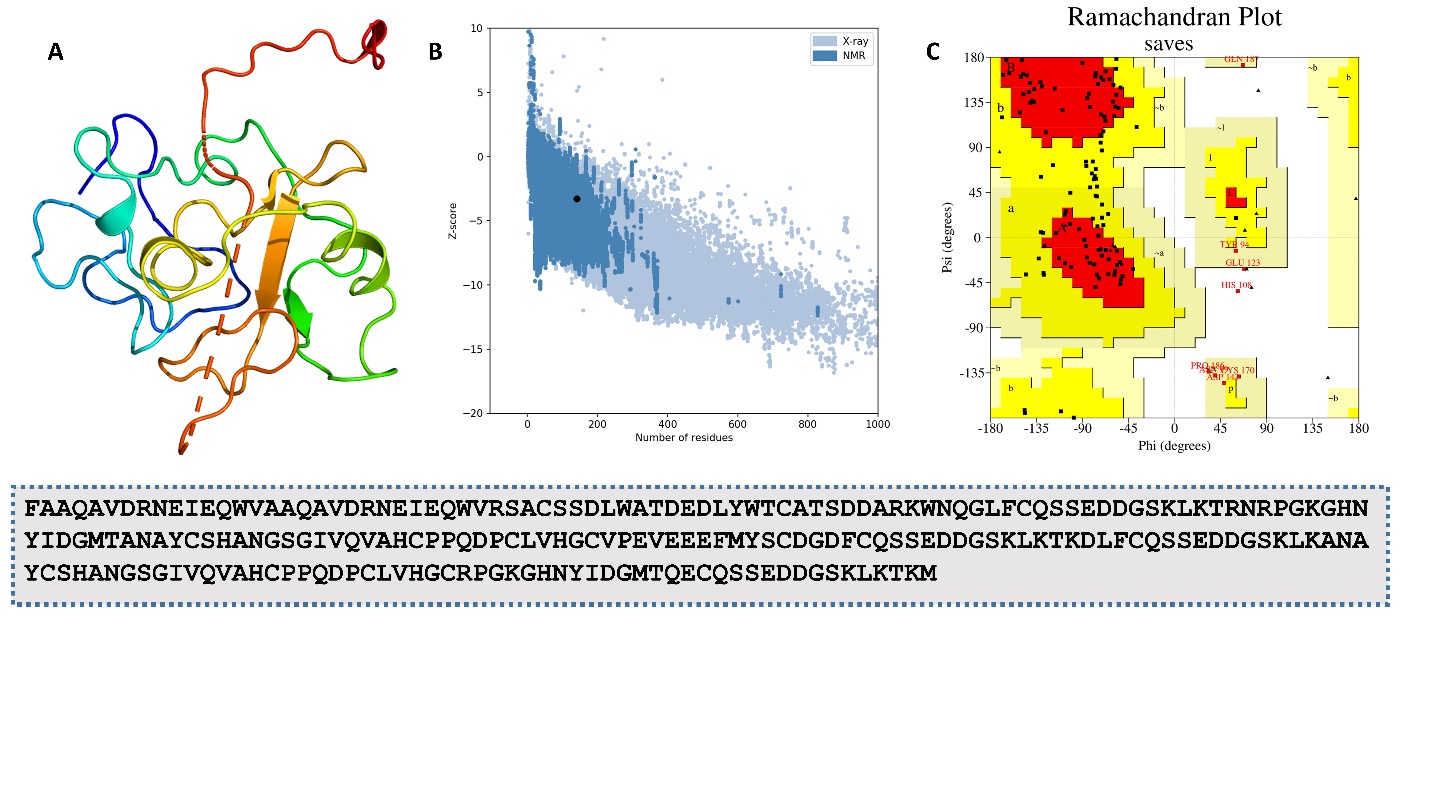
**Figure S4:** Showing structural validation of each MEVC designed against RVFV through Prosa-web. **(A-D)** represents the Prosa-web analysis of MEVCs designed against each target protein i.e. NP, L, NSP and GP, respectively.

**Figure S5:** Showing structural determination and analysis of each MEVC-PW-NEG (Negative control). **(A)** the predicted 3D structure of the negative control, **(B)** represents the Ramachandran plot of negative control MEVC designed against whole proteome, **(C)** represents the Prosa-web analysis for MEVC designed against whole proteome of RVFV.

**
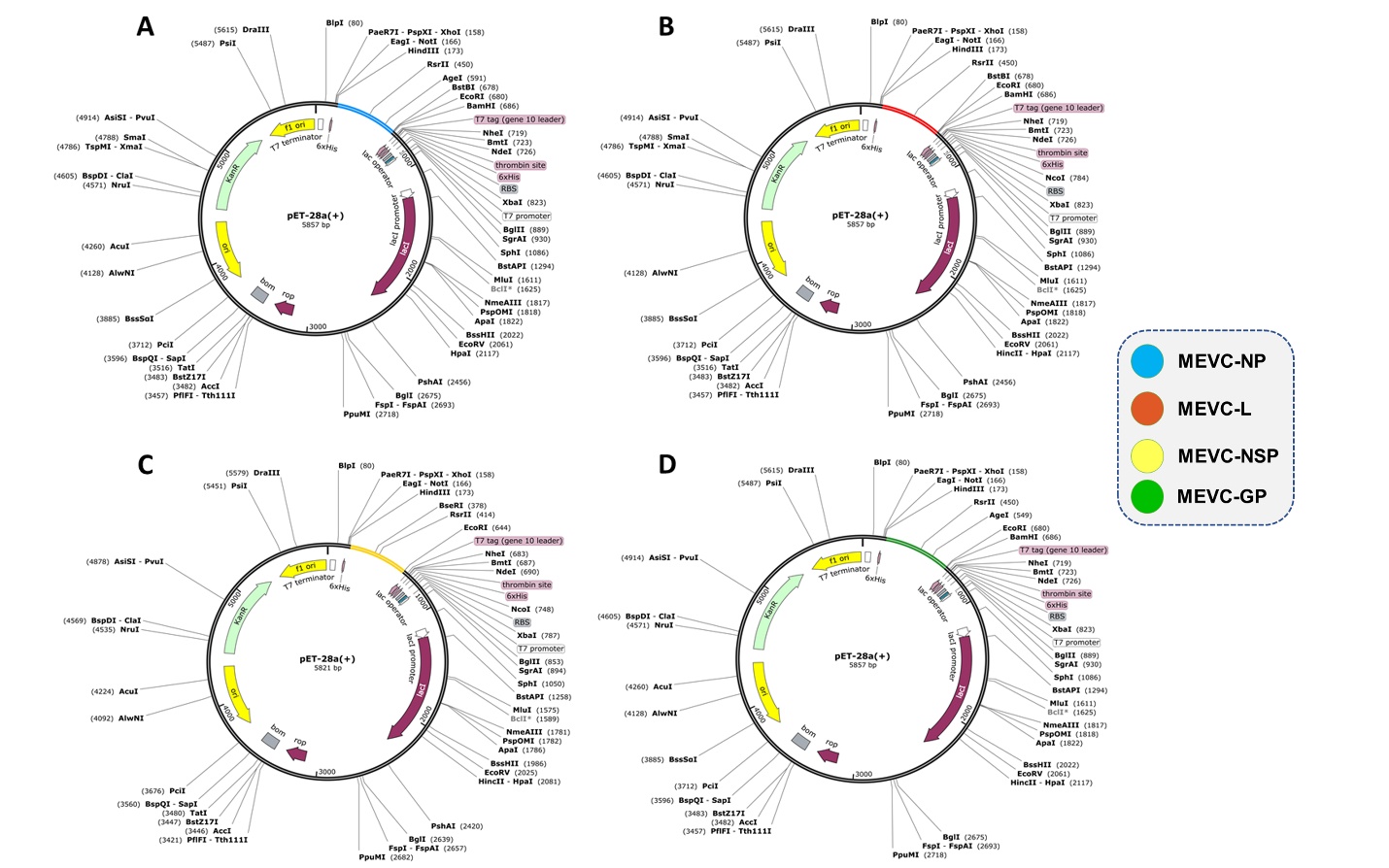
**

**Figure S6.** Showing constructed plasmid maps with differently colored inserts for each of the protein specific designed MEVC **(A-D)** represents the plasmid maps for MEVCs designed against each target protein i.e. NP, L, NSP and GP, respectively.

**
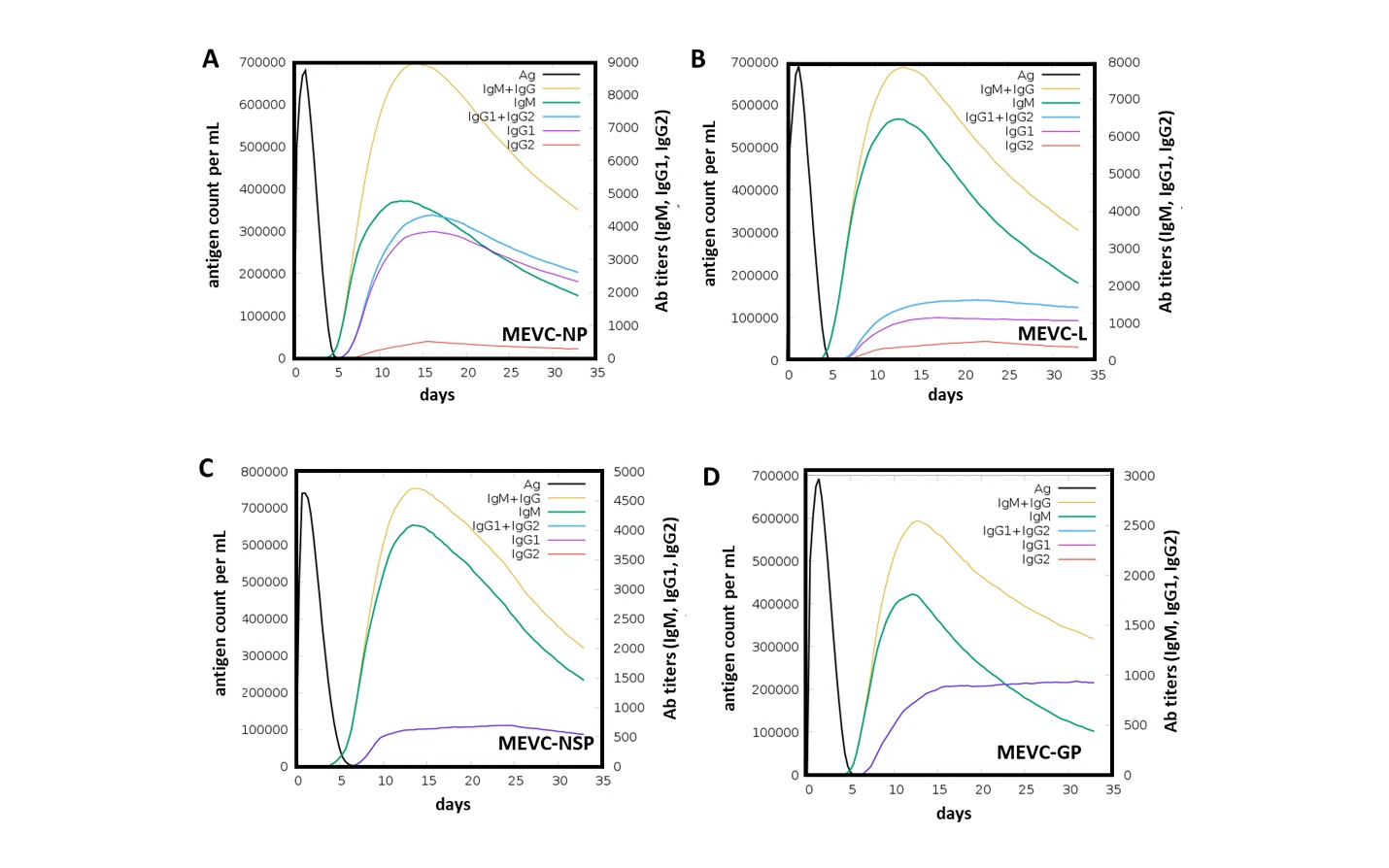
**

**Figure S7.** Showing graph-based representation of induced immune response in the form of Ab titers observed against each of the designed protein specific MEVC **(A-D)** represents the immune-simulation graphs for MEVCs designed against each target protein i.e., NP, L, NSP and GP, respectively.


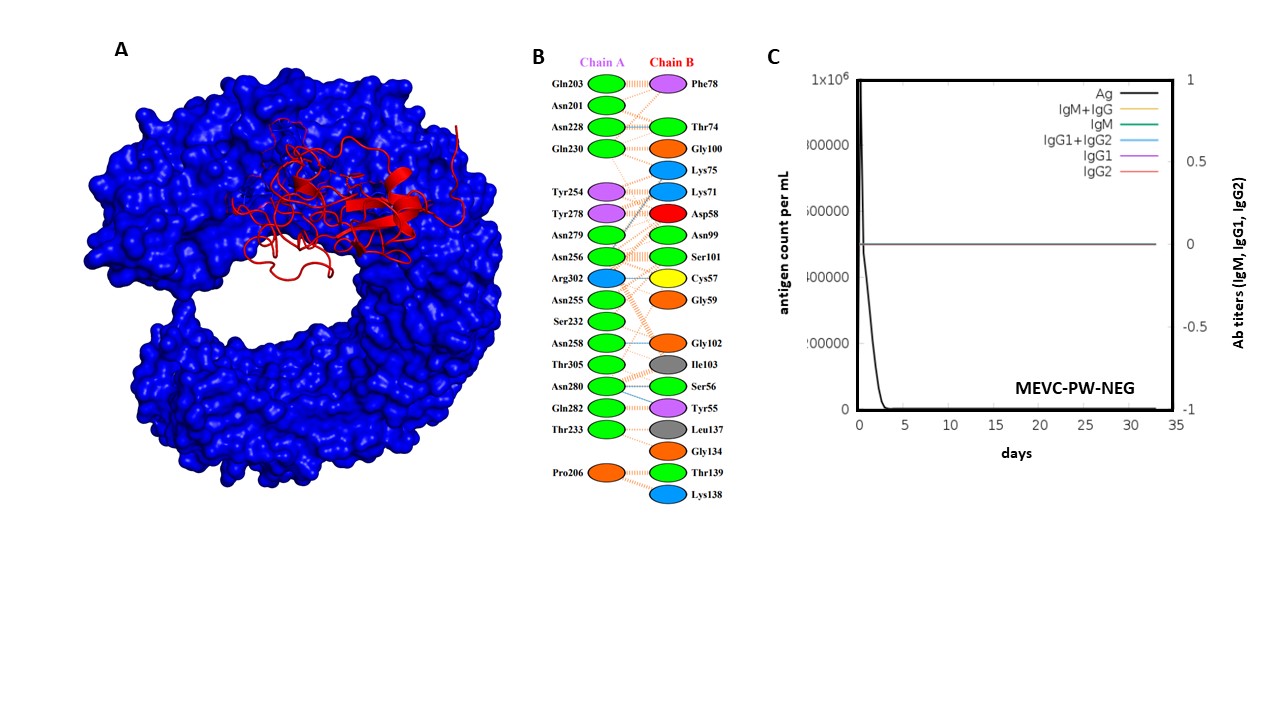


**Figure S8:** Showing docking complex analysis of MEVC-PW-NEG (Negative control) with human TLR8. **(A)** shows the analyzed 3D modelled docking complex structure of the negative control, **(B)** represents the interaction patterns of the designed MEVC (Negative control) and human TLR8 docking complex. While **(C)** represents the immune simulation graph obtained for the negative control.


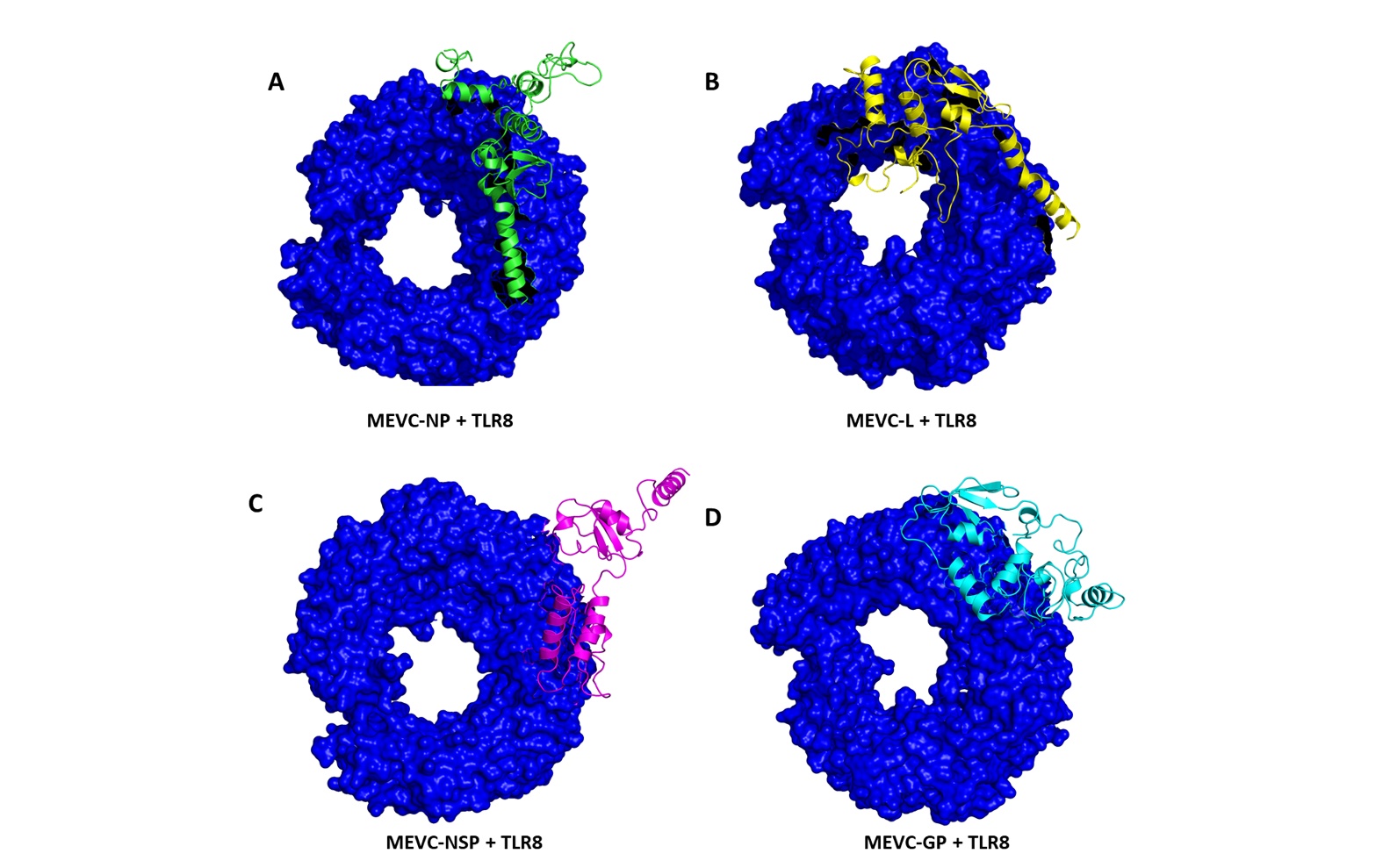


**Figure S9.** Showing structural representation of different docking complexes with human TLR8 and each of the designed protein specific MEVC **(A-D)** represents the docking complex for MEVCs designed against each target protein i.e., NP, L, NSP and GP, respectively.


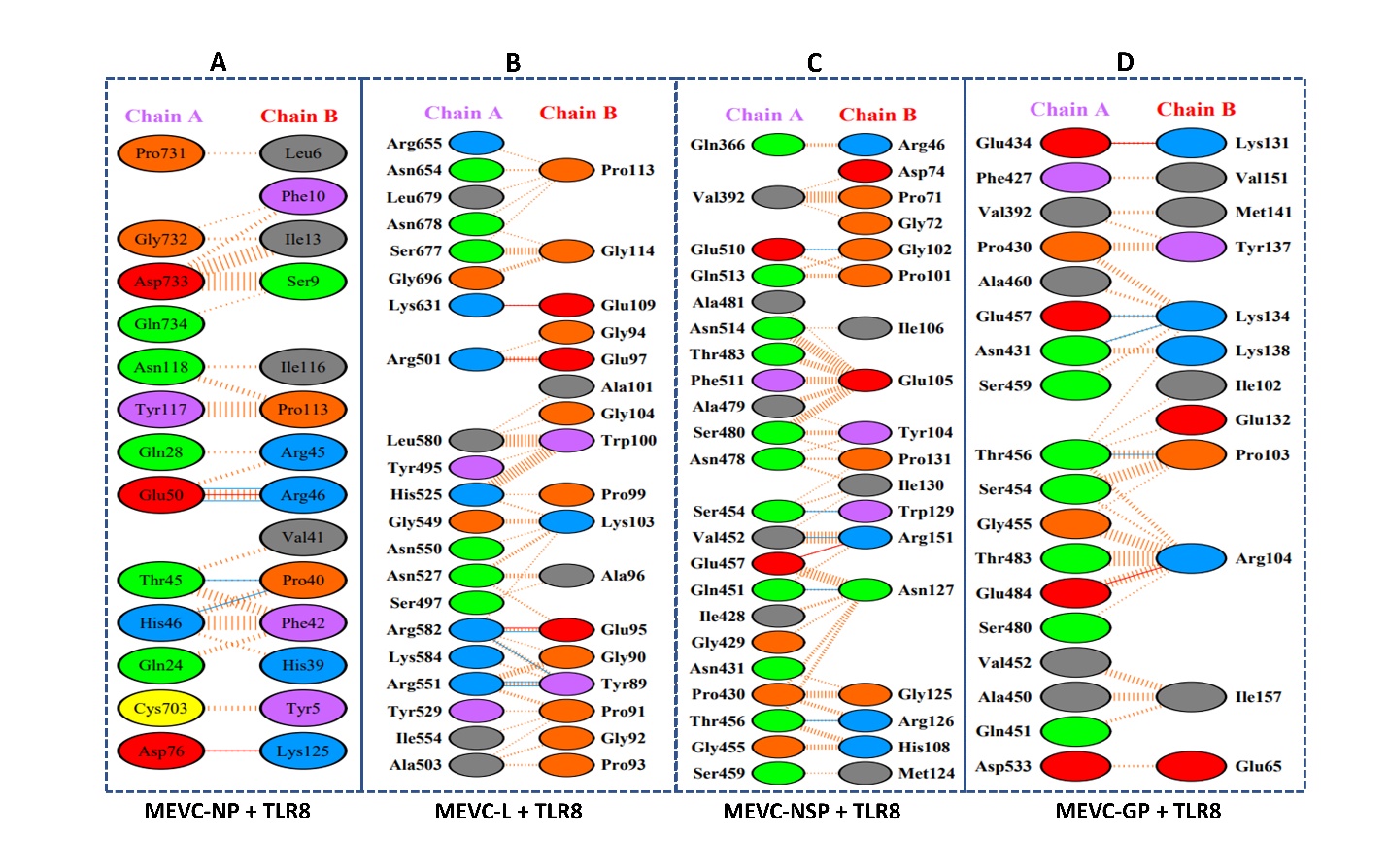


**Figure S10: (A**-**D)** Shows the performed interaction pattern analysis for each docking complex (CHAIN A represents TLR8, while Chain B represents each protein specific MEVC).
